# Supplementary material for: Three-dimensional reconstruction and the phylogeny of extinct chelicerate orders
Source: PeerJ. 2014 Nov 13;2:e641. doi: 10.7717/peerj.641 (PMC4232842; doi:10.7717/peerj.641)
Supplement: File S1 — Morphological characters statements for the 192 characters used in the current analysis, modified after Pepato, da Rocha & Dunlop (2010). [file peerj-02-641-s001.docx]

# Three dimensional reconstruction and the phylogeny of extinct chelicerate orders: Supplementary Information

Russell Garwood

Jason Dunlop

## Character statements

Here we present character statements for the current analysis, modified after Pepato, da Rocha & Dunlop (2010).

###

### Cephalic/prosomal region

1. *Head shield segments* (0 = five [cephalosoma/proterosoma]; 1 = seven [prosomal shield]).

This character refers to the number head segments (or post-acronal segments if the acron existed) included under the anterior prosomal sclerite. Pycnogonida and some Arachnida (in particular Acariformes among the mites, Solifugae, Palpigradi and Schizomida), appear to retain the original euarthropod head *sensu* Walossek & Müller 1998. We consider the “sejugal furrow” and the gap between anterior coxae I-II and posterior coxae III-IV as evidence for existence of such a separate tagma in Acariformes. We subsume Shultz’s (2007a) character 7 of the presence/absence of demarcation lines between the pro-, meso- and metapeltidium into this character.

2. *Ophthalmic ridges* (0 = absent; 1= present)

Extant Xiphosura present a pair of longitudinal crests passing near the region of the lateral eyes or equivalent area when lateral eyes are not evident. Similar structures are present in *Plesiosiro* (Dunlop 1999, description herein) and ‘non-hypoctonid’ Thelyphonida (*Mastigoproctus*; Rowland & Cooke 1973).

3. *Pleural margin* *of prosomal shield* (0 = absent; 1 = present).

The broad head shield of Xiphosura with its wide pleural margins has traditionally been treated as the plesiomorphic condition relative to arachnids (e.g. Shultz 1990, character 2); although this was largely based on using trilobites and other arachnomorphs as outgroups. If Pycnogonida or indeed megacheiran taxa are used to polarise the character for euchelicerates, the wide head shield of Xiphosura could alternatively be treated as derived.

4. *Cardiac lobe* (0 = absent; 1 = present).

Extant Xiphosura express a cardiac lobe, a feature shared with several fossil species including members of *Weinbergina*, Eurypterida, and *Chasmataspis*.

5. *Prosomal repugnatorial glands* (0 = absent; 1 = present).

The presence of these glands producing a noxious secretion is a convincing autapomorphy of Opiliones ( e.g. Giribet et al. 2002, character 12).

6. *Cucullus* (0 = absent; 1 = present).

This unique, hinged plate covering the mouthparts but of indeterminate function is a convincing autapomorphy of Ricinulei. Females have been observed using the cucullus to hold their eggs.

7. *Sternal region* (0 = broad; 1 = narrow anteriorly; 2 = narrow posteriorly; 3 = narrow throughout).

Irrespective of whether a sternum is present, chelicerates vary in the degree to which the coxae are consolidated together on the ventral surface of the prosoma. Coding follows Shultz (2007a, character 12), except that the sternal area in all acariform mites should be considered narrow (Alberti 2006). Following Shultz (2007a) we consider the abutting of the coxae themselves and *not* their endites. The presence/absence of explicit gnathobases is coded as another character.

8. *Prosomal sternum* (0 = undivided; 1 = divided).

The sternum of Palpigradi, Amblypygi, Thelyphonida and Schizomida - plus the extinct pahalgniotarbids and haptopodids - is divided into multiple sclerites. Other arachnids which have a sternum have only a single, undivided sclerite. Not applicable to taxa without a sternum.

9. *Cephalic doublure* (0 = absent; 1 = present).

In many trilobites and other Arachnomorpha, the cephalic exoskeleton continues onto the ventral side as a deflexed rim or doublure. The prosomal shield folds in on itself where the chelicerae emerge in Thelyphonida and palaeocharinid Trigonotarbida. This character is coded as ambiguous for the former.

10. Prosomal shield with lines demarcating meso- and metapeltidium (0 = absent; 1 = present).

Scorpiones and some Opiliones have lines on the prosomal shield demarcating three zones - the pro-, meso- and metapeltidium. This character is not applicable to taxa lacking a prosomal shield.

11. *Genal spines* (0 = absent; 1 = present).
Two non-arachnid chelicerates - Xiphosura and Chasmataspidida - and also Trilobita, possess genal spines. These posteriorly directed lateral extensions of the cephalic region are only seen in marine (usually bottom-dwelling) species.

###

### Mouth and pharynx

12. *Proboscis* (0 = absent; 1 = present).

A proboscis formed from three antimere elements terminating in a Y-shaped mouth (Dencker 1974) is autapomorphic for Pycnogonida (e.g. Weygoldt & Paulus 1979, character 58). Attempts to homologise it with arachnid mouthparts have largely been proved unsuccessful – see comments in Dunlop & Arango (2005) – supporting its interpretation as a unique feeding adaptation for sea spiders.

13. *Mouth* (0 = directed anteroventrally; 1 = directed posteroventrally).

The mouth of Xiphosura points backward towards the gnathobases. This condition has been interpreted as plesiomorphic for Chelicerata. Several fossils appear to have a similar backward flexure of the digestive tube – e.g. trilobites – as indicated by the backward direction of the hypostome or remnants of the gut contents. Here we score pycnogonids as 0/1, since their mouth orientation is largely dependant on the form and orientation of the proboscis (see above).

14. *Labium/tritosternum* (0 = absent; 1 = present).

The labium, or tritosternum in some terminologies, is a separate sclerite generally forming the lower lip of the mouth. Shultz (2007a) considered it present in Palpigradi, Araneae, Amblypygi, Thelyphonida, Schizomida, Trigonotarbida, Ricinulei, and in some acariformes among the mites. In fact, the labium in Palpigradi does not share the same relative position when compared to other Arachnida. Traditional studies of morphology regard the palpigrade labium as a protosternum, i.e. associated with the cheliceral segment (Börner 1902, Snodgrass 1948). Palpigradi is therefore scored 0 here for this character. Pseudoscorpions and Solifugae have a narrow medial sclerite related dorsally to the palpal coxal process. In Pseudoscorpionida, it is known as the so-called lophognath. It is crested and fits in the grooved ventral surface of the epistemolabral plate, or trophognath (Snodgrass 1948). A similar elongated sclerite may be found in some Endeostigmata, e.g. *Orthacarus* *tremli* Zakhvatkin, 1949 (Bimichaelidae; Jesionowska 2003). Because of its position, this sclerite is regarded as a deuto- or protosternum and thus not homologous to the labium as it is considered here. For a similar reason we exclude a ‘labium’ from phalangid Opiliones (Shultz & Pinto-da-Rocha 2007). We regard this as more likely to be a sternapophysis associated with first leg rather the pedipalp (Winkler 1957).

15. *Epistomal-labral plate* (0 = absent; 1 = present).

The labrum is fused to the epistome in Solifugae, Pseudoscorpiones and Acari (see also Snodgrass 1948, *contra* Shultz 2007a). The whole structure protrudes noticeably between the chelicerae and is flanked by a pair of so-called lateral lips (Hammen 1989; Dunlop 2000a). The plate and lips are here scored together as a single character complex. The plate itself is sometimes referred to as a ‘beak’ or ‘rostrum’, especially in the solifuge literature.

16. *Ventroposterior wall of pre-oral chamber* (0 = formed by labium; 1 = formed by palpal coxae).

This specific morphology of the epipharyngeal sclerite was proposed by Shultz (1990, character 5) as a putative synapomorphy of Pedipalpi. Its condition in other arachnids without a labium (see previous character) was not discussed and we score such taxa here as (?). This highlights a general problem with many of the putative skeleto-muscular synapomorphies proposed for Pedipalpi, namely that they are sometimes hard to assess across *all* arachnids and their outgroups.

17. *Stomatheca* (0 = absent; 1 = present).

This character was defined by Shultz (2007a) as a preoral chamber formed by the lateral sides of the palpal coxae and ventrally by extensions of the coxae of leg 1 and to a lesser extent leg 2. Shultz treated it as a synapomorphy of Scorpiones and Opiliones, although it has been criticised (Dunlop 2010), not least because it seems to be absent in stem-group (fossil) scorpions in which the coxae lack clearly developed apophyses. Shultz (2007a) speculated that early fossil scorpions may have had a stomatheca formed from soft lips in place of sclerotised projections, but the material available neither supports nor rejects this supposition. This is reflected in the coding of the Silurian species *Proscorpius osborni* (Dunlop et al. 2008), the Devonian *Palaeoscorpius devonicus* (Kühl et al. 2012). This character is also coded herein as absent in the fossil species *Hastocularis argus* (Garwood et al. 2014), which bears small coxapophyses on only the palpal and second walking leg coxae - such growths are entirely absent on leg 1, further supporting a convergent development of this character in Scorpiones and Opiliones.

18. *Ingestion of solid material* (0 = present; 1 = absent).

Most arachnids do not ingest solid material. Xiphosura possess gnathobases and a muscular gizzard suited for macerating solid food. There is no evidence for liquid feeding in non-arachnid fossils, and it is quite common to observe sediment (Hou & Bergström 1997) and even prey hard parts (e.g. within *Sidneya*; Bruton 1981) among the gut remains. Pycnogonida have a pharyngeal filter apparatus that certainly precludes the intake of anything larger than subcellular material (King 1973, Fahrenbach & Arango 2007). The latter authors also described 180–220 small salivary glands per jaw of *Ammothea* *hilgendorfi* - indicative of primarily liquid material intake. Digestion occurs largely as a result of salivary glands and musculature within the proboscis and oesophagus, and accordingly we have coded ingestion of solid material as absent for extant pycnogonid taxa. Several Opiliones and some mites (Opiliacarida, Oribatida, some Endeostigmata and free-living Astigmata; Pinto-da-Rocha, Machado & Giribet 2007, Walter & Proctor 1998) consume solid particles of food, although all of them have a well-developed preoral chamber so exhibit a certain degree of extraintestinal digestion. All other arachnids are liquid feeders and apparently digest their food preorally, often using specialised filtering devices (see e.g. character 20) to hinder the uptake of particulate matter. Finally, for Paleozoic scorpions this character is uncertain given that they seem to lack a well-developed pre-oral chamber (see above).

19. *Palate plate* (0 = absent; 1 = present).

This specific modification of the dorsal pharynx wall with fringed platelets used as filters to trap particulate matter from the preorally digested food is an autapomorphy of Araneae; e.g. Giribet et al. (2002, character 159).

20. *Filtering preoral setae* (0 = absent; 1 = present)

In Ricinulei and in at least *Palaeocharinus* among the Trigonotarbida there is a similar-looking filtering structure in front of the mouth consisting of either downward-pointing setae or platelets. This feature is a potential synapomorphy of these arachnids.

21. *Three-branched epistomal skeleton* (0 = absent; 1 = present).

This specific form of the epistome skeleton with three processes for the pharyngeal dilator muscles was described in detail by Shultz (2000) who proposed it as a putative synapomorphy of (Scorpiones + Opiliones).

22. *Intercheliceral epipharyngeal sclerite* (0, absent; 1, present).

Coding follows Shultz (2000, character 191).

23. *Epipharyngeal sclerite large, projecting posteriorly* (0, absent; 1, present).

Coding follows Shultz (2000, character 192). Inapplicable to taxa lacking an epipharyngeal sclerite

### Segmentation, tagmosis and telson

24. *Metasoma* (0 = absent; 1 = present).

Cotton & Braddy (2003) defined this character as a “post-abdomen lacking appendages”. This definition is hard to apply in most chelicerates since some lack recognizable abdominal appendages altogether (e.g. Palpigradi, Opiliones, Pseudoscorpiones, Acari). We seek to redefine this tagmosis character here as a posterior, limbless set of segments, typically with a cylindrical exoskeleton which is, to a greater or lesser extent, set off from the mesosoma by a narrowing of the body.

25. *Prosoma and opisthosoma form a single functional unit* (0 = absent; 1 = present)

In Opiliones and some mite taxa, the prosoma and opisthosoma fuse form a single unit. After Legg, Sutton & Edgecombe (2013) character 602.

26. *Metasoma length* (0 = three segments; 1 = five segments; 2 = nine segments).

This character is inapplicable for taxa which do not express a metasomal tagmosis.

27. *Well-developed post-anal tels*on (0= absent; 1= present).

Given that various potential outgroups among early Palaeozoic arthropods have a post-anal telson, its presence in Xiphosura, Scorpiones, Palpigradi, Thelyphonida and Schizomida is probably plesiomorphic for Chelicerata. No Recent sea spiders have a telson, but some fossil taxa do, including *Palaeisopus* (Vilpoux & Waloszek 2003); see also Walossek & Müller 1998 for discussions of ground patterns.

28. *Flagellate telson* (0 = absent; 1 = present).

In Palpigradi, Thelyphonida and (albeit in a shortened form) Schizomida, among the extant orders, and now Uraraneida, among the fossils, the telson is subdivided into multiple articles to form a distinctly flagellate, whip-like structure. Not applicable to taxa without a telson (see above).

29. *Telson with vesicle and aculeus* (0 = absent; 1= present).

This feature is regarded here as an autapomorphy of Scorpiones.

30. *Specialized male postanal flagellum* (0 = absent; 1 = present).

This modified male flagellum plays an important role during courtship – the female holds onto the male flagellum and is pulled over a spermatophore – and is widely regarded as a convincing autapomorphy of Schizomida.

### Chelicerae or deutocerebral appendage

31. *Number of chelicerae articles* (0 = more than three, 1 = three; 2 = two).

Solifugae, Pseudoscorpiones, Ricinulei and the Terapulmonata *sensu* Shultz (1990) have only two cheliceral articles. This is widely accepted as the apomorphic condition compared to the three articles seen in other (euchelicerate) taxa. Indeed, gene expression data now suggest that the presence of two articles could have arisen through loss of developmental domains along the proximo-distal axis of the appendage (Sharma et al 2012). Acariformes is scored here as having two articles. Those supporting the hypothesis that Acariformes have a proximal trochanter in the chelicerae argued that the proximoventral region of the fixed digit is a fused remnant of this article. This is based on the attachment of the cheliceral retractor muscles in this region (Evans 1992), and developmental studies support this suggestion in one species (Barnett and Thomas 2013). At least one pycnogonid species has been figured with four cheliceral articles (see e.g. Dunlop & Arango 2005) - including the fossil species included herein. The Megacheiran taxa included in the current analysis both have a deutocerebral great appendage comprising more than three articles (Tanaka et al., 2013; Haug et al. 2012), a state also seen in the synziphosurine taxa *Dibasterium durgae* (Briggs et al. 2012) and *Offacolus kingi* (Sutton et al. 2002). This outgroup choice accordingly implies the possession of more than three articles in the deutocerebral appendage the plesiomorphic state for the chelicerates.

32. *Presence of elbowed chelicerae* (0 = absent, 1 = present).

In some taxa with a three-segmented chelicera (e.g. Palpigradi and Opiliones), there is a geniculate joint between the forward-projecting the basal cheliceral element and the distal two elements (forming the claw). This arrangement allows the claws to move in the proximity of the mouth. By contrast, in groups like scorpions all three cheliceral elements simply project forwards. We do not consider the joint in the pycnogonids geniculate: the proboscis limits the required range of motion in the chelifores, which have a greater variability in orientation and podomere proportions than observed in arachnid taxa. Scored as inapplicable for those taxa with only two cheliceral articles, or more than three.

33. *Position of the cheliceral apotele* (0 = articulates ventrally; 1 = articulates dorsally; 2 = articulates laterally).

In Solifuges, Pseudoscorpiones and both major groups of Acari the distalmost cheliceral segment (the apotele) articulates ventrally against the preceding article (e.g. Dunlop 2000). In Tetrapulmonata and Ricinulei it is more or less dorsal (keeping in mind the torsion of the chelicerae in labidognath spiders). Three segmented chelicerae with an ‘elbowed’ articulation, such as in harvestmen and palpigrades, do not fit comfortably into either of these schemes and are tentatively coded as a separated character state.

34. *Cheliceral ‘fang’* (0 = chelate; 1 = ‘clasp-knife’ type; 2 = Prostigmata styliform or ‘*Anystys*’-like chelicerae).

So-called ‘clasp-knife’ chelicerae, *sensu* Shear *et al*. (1987), in which the apotele forms a fang rather than the movable finger of an explicitly chelate claw, are found in Araneae, Uraraneida, Amblypygi, Thelyphonida, Trigonotarbida and Schizomida (see also Kraus 1976). The chelicerae of Ricinulei also approach this condition (see e.g. Dunlop 1996, fig. 11) with a longer ‘fang’ articulating against a shorter fixed tooth and are thus scored 0/1 here for this character. Several Prostigmata also have the fixed digitus reduced, but the movable digit is ventral and the condition is clearly non-homologous to the condition observed in Tetrapulmonata and is scored separately here.

35. *Naked cheliceral fang* (0 = absent; 1 = present).

Selden, Shear & Sutton (2009) suggested that the loss of setae (or other similar sorts of projections) on the cheliceral fang was an autapomorphy of Araneae. Selden, Shear & Bonamo (1991) and Selden, Shear & Sutton (2009) demonstrated a naked fang in *Attercopus*; i.e. this character would support (Uraraneida + Araneae). The fang (or movable finger) in other arachnids seems usually to be setose and/or dentate. The condition observed in some mites is not considered as primarily homologous to this character.

36. *Plagula ventralis* (0 = absent, 1 = present).

This specific small sclerite between the fang and the basal segment of the chelicera has been proposed as a synapomorphy of Araneae, Amblypygi, Thelyphonida and Schizomida (Homann 1985).

37. *Cheliceral venom gland* (0 = absent; 1 = present).

Traditionally treated as an autapomorphy of Araneae, venom glands opening through the movable finger (or fang) of the chelicerae are present in all the spiders scored here. Venom glands are also absent in the ingroup spider family Uloboridae (not scored here) and while there have been claims that they are absent in the basal Mesothelae spider clade, this proposal has since been refuted. As a result of the observation of a venom gland in *Attercopus*, this character also supports a (Uraraneida + Araneae) clade.

38. *Endocephalic spinning apparatus* (0 = absent; 1 = present).

The endocephalic spinning apparatus, together with its associated spinnerets or galea, is an autapomorphy of Pseudoscorpiones (Harvey 1992). Whether these glands – which open on the movable finger of the chelicerae (Weygoldt 1969) – are homologous with the venom gland of spiders is unclear.

39. *Cheliceral flagellum* (0 = absent; 1 = present).

This sometimes complex projection from the dorsal surface of the fixed finger of chelicerae in male Solifugae can take a number of forms, but is (secondarily) absent in the solpugid family Eremobatidae (Punzo 1998). Its precise function in solifuges is not well understood. The character has been treated as an autapomorphy of this order. However, Harvey (1992) regarded the cheliceral flagella of Solifugae and Pseudoscorpiones (see e.g. Weygoldt 1969, fig. 2) as potentially synapomorphic and this is reflected in the scoring here. Notice that the flagellum occurs on the fixed finger and should *not* be confused with the galea which is on the movable finger of the pseudoscorpion chelicerae (see previous character).

40. *Chelicerocarapacal articulation* (0 = absent; 1 = present).

A specific antero-lateral articulation between the prosomal shield and the basal cheliceral article has been described from Solifugae and (most) Pseudoscorpiones, and suggested as a potential synapomorphy for these orders; see e.g. Shultz (1990, character 13).

41. *Mesal fusion of chelicerae* (0 = absent; 1 = chelicerae proximally fused).

Mesal fusion of the chelicerae is seen in mites of the following groups: Tetranychoidea, Raphignathidae, Caligonellidae, Tarsonemina, and in some Tydeidae and Stigmaeidae (Evans 1992). A similar arrangement can also occur (certainly secondarily) in some spiders such as Filistatidae, but not among the taxa scored here.

42. *Movable digit* (0 = ‘*Anystis*’ type; 1 = styliform).

Among those Acariformes with a reduced digitus fixus, two conditions may be observed: the mostly curved, dorsally serrate digits of Trombidiidae, Halacaridae, Trombiculidae, Anystidae and Paratydeidae on the one hand and a smooth movable digit of Erythraeidae, Smariididae, Cheyletidae and Tetranychidae, suited for piercing, on the other. This character is only applicable to mites without the digitus fixus (cf. character 36, state 2).

###

### Pedipalps or second head appendage

43. *Palpal coxae* (0 = free; 1 = fused medially).

This is a somewhat problematic character in that it is potentially part of the same character complex which embraces the gnathosoma of mites and, perhaps, ricinuleids (see below). Irrespective of the gnathosoma, a straightforward medial fusion of the pedipalpal coxae is seen in both major groups of Acari, as well as in Ricinulei, Thelyphonida and Schizomida. In the latter two orders it is usually referred to explicitly as a camerostome; see e.g. Yoshikura (1975, table 24) and Shultz (1990, character 18).

44. *Gnathosoma* (0 = absent; 1 = present).

The gnathosoma essentially consists of the fused palpal coxae (see previous character) forming the subcapitulum, plus the chelicerae and mouth lips. All these elements articulate together as a single movable unit against the rest of the body. It has been cited (Lindquist 1984) as one of the most convincing characters for defining mites as a monophyletic group, although its homology was challenged by Hammen (1989) who noted significant differences in the patterns of muscular insertion (see also Alberti 2006). Confusingly, some authors also recognise a gnathosoma as present in Ricinulei; thus essentially the same character has been used by different authors to support either a monophyletic Acari or (Acari + Ricinulei). We find the latter interpretation problematic, and followed here Shultz (2007a) in coding this as ambiguous.

45. *Subcapitular rutella* (0 = absent; 1 = present).

These modified, thickened setae are found in basal members of both Parasitiformes and Acariformes among the mites. Although absent in more derived Anactinotrichida and often cited as lost in prostigmatid mites, they were noted as present in Rhagidiidae by Zacharda (1980) although the structures observed in Rhagidiidae and scored here do not resemble the Oribatida or Opilioacarida rutella. Irrespective of ingroup reversals, rutella appear to be one of the more convincing characters defining Acari as a monophylum (Lindquist 1984, character 1). Alberti (2006, and references therein) has, however, questioned how well this character is understood and cautioned about accepting its homology in all groups where it occurs.

46. *Palpal chelae* (0 = leg like; 1 = subraptorial; 2 = chelate; 3 = ‘scorpionid’; 4 = ‘thumb and claw’).

Large, subraptorial pedipalps characterise Amblypygi, Thelyphonida and Schizomida. The pedipalps or equivalent appendages of Xiphosura, Ricinulei and at least *Palaeocharinus* and *Anthracomartus* among the Trigonotarbida, end in small terminal claws or pincers in which the apotele opposes a corresponding projection from the tarsus. Scorpiones and Pseudoscorpiones share a specific and potentially homologous ‘scorpionid’ claw morphology involving a large manus, containing a similar musculature, and elongate fingers; see e.g. Shultz (1990, characters 16 and 17) for details. Several Prostigmata among the mites have a strong ‘claw’ (in fact, a hypertrophied seta) in the palptibia which acts against the palpal tarsi. This ‘thumb and claw’ organisation is seen in Tetranychoidea, Stigmaeidae, Cheyletidae, terrestrial Parasintegona, Erythreaoidea, and Anystidae among the taxa scored here.

47. *Palpal cleaning organ* (0 = absent; 1 = present).

This specialised brush of two highly organised rows of tarsal setae (e.g. Weygoldt 2000, figs 133-139) has been called the ‘cleaning organ’ or ‘cleaning brush’. It is one of the few explicit autapomorphies of Amblypygi (Shear et al. 1987).

48. *Pedipalpal venom glands* (0 = absent; 1 = present).

Poison glands within the palpal chelae – which can open in one or both fingers (Weygoldt 1969) – represent a convincing apomorphy of the ingroup pseudoscorpion clade Iocheirata *sensu* Harvey (1992)/Murienne, Harvey & Giribet (2008). It should be reiterated that venomous pedipalpal claws are not an autapomorphy of *all* Pseudoscorpiones.

49. *Palpal apotele* (0 = differentiated from tarsus; 1 = not differentiated from tarsus).

Although proposed by Shultz (1999, character 14) as a putative synapomorphy of Pedipalpi only, it is also applicable to the large claws making up the pedipalps of Scorpiones and Pseudoscorpiones too. Apoteles are also absent from the palps of Acariformes and have been convergently lost in at least some Opiliones, such as the genera *Nemastoma* and *Sabacon* (Alberti 2006; Giribet et al. 2002).

50. *Adhesive palpal organ* (0 = absent; 1 = present).

An adhesive structure at the end of the pedipalp (Punzo 1998) is a putative autapomorphy of Solifugae. Given that solifuges do not express a palpal apotele *sensu stricto* (but see previous character) and that the palpal organ articulates via lateral condyles, it could potentially represent a highly modified claw (Dunlop 2000b).

### Ovigers and legs

51. *Ovigers* (0 = absent; 1 = present).

Modification of the third limb into an egg-carrying oviger is an autapomorphy of the ground pattern of Pycnogonida Giribet et al. (2002, characters 11,40). However, like the chelifores and pedipalps, ovigers are secondarily reduced in some derived ingroup sea spiders – for example *Endeis* lack ovigers completely – as well as females of Pycnogonidae and Phoxichilidiidae (Arango 2002).

52. *Gnathobases* (0 = present; 1 = absent).

Among recent Chelicerata, only Xiphosura retains dentate gnathobases along all postcheliceral limb coxae. This is widely accepted as a plesiomorphic mode of feeding. Among fossils they are present in several taxa, including trilobites, eurypterids, various arachnomorphs, and the outgroup taxon -

*Alalcomenaeus* - in the current analysis, which implies that gnathobasal feeding is at some level the original mode of ingestion. Serrula on the pedipalpal coxae (or gnathocoxae) of spiders have to some extent ‘reinvented’ the masticatory function of the gnathobases, but in detail they differ from the dentate xiphosuran/eurypterid gnathobase and are not scored as homologous here.

53. *Antenniform first leg* (0 = absent; 1 = present).

This antenniform limb was proposed by Shultz (1999, character 16) as a putative synapomorphy of Pedipalpi. Solifugae and Palpigradi also walk hexapodally to some extent and probe ahead with the first leg. However, they do not show the same degree of structural modification of this limb as in Pedipalpi where the antenniform leg pair is markedly different from those used in walking.

54. *Leg 1 sternocoxal articulation* (0 = absent; 1 = present).

This specific pattern of articulation was proposed by Shultz (1999, character 25) as a putative synapomorphy of Pedipalpi.

55. *Apotele of first leg* (0 = present; 1 = lost).

This reduction of the apotele in these antenniform legs was suggested by Shultz (1999, character 17) as a potential synapomorphy of Amblypygi, Thelyphonida and Schizomida. The apotelic claws is retained in Amblypygi, but in a highly reduced form (e.g. Wegoldt 2000, fig. 81). The leg 1 apotele is replaced by a bush of hairs in *some* Solifugae (Roewer 1934) which are here scored 0/1 for this character.

56. *Leg 2* (0 = unmodified; 1 = elongate).

In Ricinulei and *most* Opiliones – but not in the putatively basal Cyphophthalmi – the second walking leg (or 4^th^ prosomal limb) is noticeably longer and is typically used to probe ahead of the animal (see also Giribet et al.2002, character 70).

57. *Exopods* (0 = retained on more than one prosomal limb; 1 = on sixth prosomal limb, 2 = lost).

From the original biramous limb, the exopod is retained as the so-called flabellum on the coxa (or basipod in some terminologies) of the last pair of legs in Xiphosura. The exopod on this limb is lost in all Arachnida; see e.g. Giribet et al. (2002, character 110). Dunlop, Anderson & Braddy (2003) reported a dissociated biramous leg belonging to *Chasmataspis* that resembles the ﬂabellum on prosomal limb VI of Xiphosuran. As with most Arachnomorpha, the enigmatic Herefordshire taxa *Offacolus* (Orr et al. 2000, Sutton et al. 2002) and *Dibasterium* (Briggs et al. 2012) retain biramous legs in the prosomal appendages. *Weinbergina* is reported as possesing only uniramous appendages (Stürmer & Bergström 1981; Moore et al. 2005) - although this could be worthy of restudy based on these recent discoveries from Herefordshire.

58. *Coxotrochanteral joint* (0 = simple; 1 = complex).

The coxotrochanteral joint of Araneae, Amblypygi, Thelyphonida and Schizomida has been described as being of a more complex nature – specifically through including so-called intercalary sclerites – compared to the simple bicondylar articulation seen in other taxa; cf. Shultz (1989, 1990 character 24) for details.

59. *Divided femora in legs 3 and 4* (0 = present; 1 = lost).

Shultz (1989, 1990 character 25) discussed previous interpretations of this ‘extra’ limb article which has been variously regarded either as a double trochanter or an extra femur. It was treated by Shultz as plesiomorphic retention of a basi- and telofemur; a character thus retained in Pycnogonida, Ricinulei, Solifugae and Acari. In some schemes (e.g. Hammen 1989) Palpigradi was also interpreted as having two femurs, but this assumption was not followed by Shultz (1989) whose interpretations based on musculature we rely on here for scoring the character.

60. *Femoropatella articulation* (0 = transverse hinge; 1 = bicondylar articulation; 2 = monocondylar articulation).

Interpreted by Shultz (2007a, character 69) as a hinge in most arachnids, the bicondylar condition occurs in Opiliones, Scorpiones and Pseudoscorpiones. A monocondylar articulation here appears to be autapomorphic for Solifugae.

61. *Patellotibial articulation* (0 = monocondylar; 1 = hinge; 2 = bicondylar).

The monocondylar condition of this joint was assumed (Shultz 1990, character 31) to be plesiomorphic, with modification to a hinge in Acari, Ricinulei and Solifugae and to a bicondylar structure with an additional ventral articulation in Opiliones, Scorpiones and early derivative Pseudoscorpiones.

62. *Patellatibial articulation with auxiliary posterior articulation* (0 = absent; 1 = present).

This specific pattern of leg articulation was proposed by Shultz (1999, character 22) as a putative synapomorphy of Pedipalpi. How this relates to the previous character is unclear and these ventral/posterior articulations may turn out to be part of a single character complex; perhaps homologous with the additional ventral articulation alluded to in character state 2 above.

63. *Appendages of postoral somites III–V with fused tibia and tarsus* (0 = absent; 1 = present).

A fused tibia-tarsus is observed in extant Xiphosura, where it forms the fixed finger of the distal claw and articulates against the tarsus as the movable finger. This arrangement is apparently not seen in the Devonian synziphosurine *Weinbergina* (Moore et al. 2005) which seems to retain the tibia and tarsus as separate elements, but is not apparent in the other included fossil taxa.

64. *Tarsus* (0 = tarsus divided into basi- and telotarsus; 1 = tarsus undivided).

The ‘basitarsus’ *sensu* Shultz is equivalent to the metatarsus in more usual arachnological terminology. State 1 occurs in extant Xiphosura (Shultz 1989) and in Acariformes (Lindquist 1984, Evans 1992), with the exception of Erythracarinae among Anystidae; a probable convergence. It also occurs in the anterior two pairs of legs in chthonioid pseudoscorpions, for which the character is coded here as 0/1, and all legs in Feaelloidea (Chamberlin 1931). A circumtarsal ring in Anactinotrichida may represent a joint between a basi- and telotarsus (Evans 1992), however we follow Shultz (2007a) in coding the tarsus as undivided.

65. *Telotarsi of walking legs 2–4 with three tarsomeres* (0 = absent; 1 = present).

This specific pattern of tarsal division was proposed by Shultz (1999, character 18) as a putative synapomorphy of Pedipalpi, but also appears to occur in the extinct order Haptopoda; these together forming a putative Schizotarsata clade Shultz (2007a). Some other arachnids, particularly various long-legged Opiliones, may show numerous tarsomere divisions at the distal ends of their legs; but not the specific arrangement scored for this character.

66. *Apotele* (0 = a simple cone or blade; 1 = a medial piece, comprising a claw or pulvillus, more frequently bearing a pair of lateral claws).

Most Arachnomorpha present a tridactyl terminal piece at the ends of their legs. The main exceptions comprise Eurypterida, *Chasmastaspis*, some fossil scorpions and extant Xiphosura (Dunlop 2002b). For outgroup *Emeraldella brocki*, this is coded as absent as the taxon has a conical, central spine surrounded by possibly movable spines - however, these do not appear to form a claw (Stein and Selden 2013).

67. *Pulvillus* (0 = absent; 1 = present).

A fleshy (?adhesive) pad between the claws of the legs has been variously named a pulvillus or empodium. This structure can be found in Pseudoscorpions, Solifugae, ‘pulvillate’ Amblypygi and Parasitiformes among the mites. When a fleshy structure similar to a pulvillus occurs among the Acariformes sampled, such as *Sancassania* or *Rhizoglyphus*, it is invariably accompanied by a medial claw.

68. *Chelate legs* (0 = absent; 1 = present).

The legs (i.e. limbs III–VI) in extant Xiphosura, *Offacolus*, *Dibasterium,* and *Chasmastaspis* (where known) end in claws formed by the subterminal apotele moving against the tarsus or tibiotarsus. Note that some of the anterior limbs in the Recent Xiphosura from SE Asia are more subchelate in nature.

69. *Divided claws* (0 = absent; 1 = present).

Subdivided claws, or ungues, on the walking legs in which the tips articulate against the rest of the claw (Roewer 1934, Weygoldt 1969, Dunlop 2000b) are a putative autapomorphy of Solifugae.

###

### Opisthosoma and opisthosomal appendages

Incomplete data on the embryology of some arachnid orders makes opisthosomal structure, and its possible appendages, one of least well-understood issues in chelicerate morphology. Almost all structures associated with opisthosomal segments have been argued as limb-derivatives at some stage. Only xiphosuran chilaria, the scorpion pectines, spider spinnerets, the book lungs, book gills, and certain spider trachea can presently be positively identified as appendage derivatives (Damen, Saridaki & Averof 2002). Our coding strategy assumes that these structures are homologous to the posterior limbs of arachnomorph arthropods; probably corresponding mainly to the exopods (but see individual characters like lungs and lung opercula for details). See discussion section of Pepato, da Rocha & Dunlop (2010) for further details of possible leg derivatives.

70. *Plate-like opisthosomal appendages* (0 = absent; 1 = present).

Weygoldt & Paulus’ (1979) defining character for Euchelicerata, these plate-like appendages, or opercula, are not seen in Pycnogonida – not even in fossil forms (e.g. *Palaeoisopus*) which retain a longer trunk behind the last walking leg. They occur as largely gill-bearing structures in Xiphosura and have been demonstrated (Shultz 1993, 1999) in the lung-bearing somites of at least the tetrapulmonate arachnids. Thus the traditional second and third ‘sternites’ of the opisthosoma are in fact highly modified, lung-bearing appendages. These probably occur in early fossil Scorpiones and may thus contribute to the sternites of modern scorpions (Jeram 2001) - a hypothesis supported by developmental studies (Farley 2005, and citations therein). The character is harder to score for those arachnids which lack lungs as it is unclear whether their sternites in the anterior opisthosomal region are true sternites, sutured on opercula or conceivably combinations of both as per the Jeram (2001) hypothesis for scorpions. Such equivocal taxa are thus scored (?).

71. Limb VI (0 = unmodified; 1 = "pusher"; 2 = paddle)

The sixth limb in arachnids is generally a pediform structure used as a walking leg. In modern xiphosurans the sixth limb has flaps near the distal end which can splay out and, similar to a snowshoe, provide extra traction when the limb is pushed down onto the substrate. It is conventionally called the ‘pusher’ as it helps push the animal forwards. Whilst *Dibasterium* possesses a specialised sixth limb, this in a single elongate, slender projection and is thus coded as unmodified for this character. In some eurypterids and chasmataspidids the sixth limb is modified into a paddle and presumably enabled the animal to swim.

72. *Number of body segments* (0 = twenty; 1 = nineteen; 2 = eighteen; 3 = seventeen; 4 = sixteen; 5 = fifteen; 6 = fourteen; 7 = thirteen; 8 = twelve; 9 - 8 segments or less).

Shultz (2007a, character 95) scored the number of opisthosomal segments. This approach works for chelicerates since the prosoma/opisthosoma boundary may be accurately determined by the presence of a reliable marker in the form of the genital opening on 2^nd^ opisthosomal segment, something absent in Pycnogonida. We followed Wills *et al*.’s (1998, character 41) approach of simply coding the total number of body segments. For Euchelicerata this means adding seven (one limbless plus six appendage bearing prosomal segments) to the number coded by Shultz. Acariformes mites often have some degree of anamorphosis, i.e. the addition of new segments during post-embryonic development. The scoring for them tries to encompass the inside group polymorphism by following Kethley’s (1990) account on this aspect. In Leanchoilid outgroup taxa we have not considered the telson a segment. Scorpions are coded as possessing twenty segments on the basis of evolutionary developmental for an additional segment, lost in adult scorpions (Simonetti 2006).

73. *Prosoma–opisthosoma junction* (0 = broad; 1 = Xiphosuran ‘cephalothorax’; 2 = narrow, pedicel).

At least the extant Xiphosura seem to have taken the trend of adding segments to the head even further. Their ‘cephalothorax’ *sensu* Scholl (1977) and Shultz (2001) incorporates dorsal elements of the 7^th^ and 8^th^ somites; a potentially derived condition giving them a longer head shield than that of the arachnid prosomal shield. This character is difficult to assess in fossil xiphosurans where embryological and musculature data are lacking. Note that this specific use of cephalothorax should be differentiated from its more traditional usage in general arachnological terminology as a synonym of prosoma.

Although some authors previously recognised a simplistic division into ‘Latigastra’, with a broad prosoma-opisthosoma junction and ‘Caulogastra’, with a narrow junction; this character proves to be rather more complex, especially when applied to arachnid outgroups. According to their incipient tagmosis, most Arachnomorpha lacks any sharp distinction between their anterior and posterior trunk segments. In early aquatic Euchelicerata like Chasmataspidida, and several Xiphosurida, the seventh tergite, or first opisthosomal tergite is distinctively narrower than the subsequent opisthosomal tergites. In other Xiphosurida and Scorpiones, the ‘broad’ connection between the prosoma and opisthosoma is probably a reversal from state 1, due to loss of most or all of the external expression of the seventh (i.e. pregenital) segment: cf. the xiphosuran microtergite and the incorporation of opisthosomal segments into its ‘cephalothorax’. This condition has been presupposed for eurypterids too (Dunlop and Webster 1999), however in the absence of developmental or muscular evidence, the junction is coded as broad here. The junction between the two body tagma is narrow in Solifugae, Palpigradi, Ricinulei and Amblypygi and Araneae. Some authors (Weygoldt & Paulus 1979) would term it the pedicel and restrict it to Araneae and Amblypygi; others prefer to consider several distinct kinds of ‘pedicel’ (Shultz 2007a, character 97). We acknowledge such difficulties of definition and prefer to recognise a more general narrowing, a probable result of the constriction of the 7^th^ segment that may have affected other segments (e.g. Ricinulei).

74. First opisthosomal tergite (0 = unmodified; 1 = very short)

This character concerns the size of the first opisthosomal tergite in comparison to those immediately posterior. Inapplicable to Pycnogonids where the first ‘opisthosomal’ segment bears a pair of walking legs. In modern Xiphosura segment one is incorporated into the prosoma, and this character has been coded inapplicable, and unknown for fossil taxa where the nature of the first segment is unknown. Present in eurypterids, haptopods, and chasmataspids, and also coded as present in the Haptopoda on the basis of Dunlop (1999) and the current work.

75. Six abbreviated opisthosomal tergites (0 = absent; 1 = present)

The extinct Phalangiotarbida uniquely express a dorsal opisthosomal surface in which the first six tergites form short bands across the back of the animal. The sixth tergite may be a little longer.

76. Opisthosomal sternite 1 (0 = present, 1 = absent)

Assuming that the lung-bearing elements on the ventral surface of Trigonotarbida are (as in Thelyphonida) the anterior and posterior opercula respectively, then studies of well-preserved trigonotarbids suggest that a first sternite is absent here, which seems to be unique for this extinct order.

77. *Locking mechanism between opisthosoma and prosoma* (0 = absent; 1 = present).

A specific ‘locking mechanism’ in which a modified first opisthosomal tergite slots into a corresponding fold at the back of the prosomal shield is observed in Ricinulei and Trigonotarbida (Dunlop 1996).

78. Opisthosomal tergites 7-10 (0 = separate; 1 = fused into a single plate)

As noted above, all Phalangiotarbida have six abbreviated tergites. Behind this there are either a series of longer tergites (e.g. *Bornatarbus*) or in some taxa, such as *Goniotarbus* addressed here, these tergites have fused completely to create a single dorsal plate covering the posterior half of the opisthosoma.

79. *Opisthosomal tergites trilobate (with a distinct axial region)* (0 = absent; 1 = present).

State 1 is found in Ricinulei and Trigonotarbida (Dunlop 1996). Coded as absent in outgroups which lack differentiated median and lateral plates for the tergites, even if trilobate in general form.

80. *Diplosegments* (0 = absent; 1 = present)

Selden (1981) noted that the three large dorsal tergites posterior to the locking structure in the fossil Ricinulei *Terpsicroton* *alticeps* are fused together, since they preserve two putative pairs of muscular attachment scars. Fusion of at least the two tergites behind the locking ridge into a single diploterite is a character apparently shared with (most) Trigonotarbida (Dunlop 1996).

81. *Fused opisthsomal segments* (0 = absent; 1 = buckler; 2 = thoracatron).

In crown-group Xiphosura – but not the fossil stem-assemblage known as synziphosurines (see e.g. Anderson & Selden 1997) – the opisthosomal tergites are fused together into a single, rigid plate termed the thoracatron or tergum *sensu* Shultz (2001). In chasmataspids three tergites are fused into a structure conventionally called the ‘buckler’ (Dunlop, Anderson & Braddy 2003).

82. *Appendages on opisthosomal segment 1* (0 = present; 1 = lost).

Modern Xiphosura retain this appendage as the chilaria and in some early synziphosurines like *Weinbergina* it may even have been retained as a fully-developed leg. At least in recent homology schemes (Vilpoux & Waloszek 2003), a limb on the first opisthosomal segment as per arachnids is also present as the last walking leg of sea spiders. Their four pairs of walking legs on segments 4–7 are, in this model, no longer serially homologous with those of arachnids (segments 3–6). Shultz (1990, character 39) proposed loss of appendages on opisthosomal segment 1 as a putative synapomorphy of arachnids, making specific reference to adult instars.

83. *Median abdominal appendage* (0 = absent; 1= present).

Eurypterids and at least those chasmataspids which are well-preserved ventrally express an often elongate and/or segmented element projecting backwards from the second opisthosomal segment; the putative site of the genital opening. For this reason this structure is conventionally referred to as the median abdominal (or genital) appendage, although it has also been referred to as a ‘Zipfel’ in some publications. It presumably plays a role in either mating or oviposition, which some debate in the literature about its gender assignment and precise function.

84. *Two anteriormost abdominal appendages fused to form genital operculum* (0 = absent; 1= present).

In eurypterids the first two pairs of mesosomal appendages are fused to form a plate called the Blatfuss/genital operculum.

85. *Embryonic appendages on 7^th^ segment* (0 = absent; 1 = present).

Scorpiones and Solifugae clearly retain limb buds on the 7^th^ (or first opisthosomal) segment during early embryogenesis (Farley 2005, fig. 1) and there are hints that the scorpion sternum may be derived (at least in part) from elements of opisthosomal segment 1 (Farley 2005, see also the next character). Unknown in fossil taxa.

86. *Genital operculum overlaps sternite of third opisthosomal segment* (0 = absent; 1 = present).

This so-called ‘megoperculate’ condition is clearly present in Amblypygi, Thelyphonida and Schizomida, whereby the second opisthosomal (or genital) operculum – see above – is quite large and overlaps a largely vestigial ‘true sternite’. This overlap was scored by Shultz (1990, character 42) as present in spiders too, but this is hard to reconcile with the fact that only Mesothelae retain the full genital sclerite (in other taxa it is reduced to a pair of book-lung opercula) and even in mesotheles evidence for the overlap of the third sternite seems to us equivocal. This is, however, tentatively coded as present. Palpigradi clearly lack a ‘megoperculum’, since the unpaired anterior lobe which accompanies the genital opening evidently does not overlap the sternite; *contra* Shultz (2007a).

87. *Opisthosomal silk glands and spigots* (0 = absent; 1 = present).

These complex glandular structures, producing silk and opening via spigots are synapomorphic for serikodiastida (Araneae + Uraraneida; Selden, Shear & Sutton 2009).

88. *Opisthosomal spinnerets* (0 = absent; 1 = present).

Spider spinnerets are appendage-derived opisthosomal structures which represent an unequivocal autapomorphy of Araneae. Specifically, they are not seen in Uraraneida in which the spigots appear to have been loosely distributed across the relevant segments of the ventral opisthosoma (Selden, Shear & Sutton 2009).

89. *Ventral sacs* (0=absent, 1 = present).

Ventral sacs are enigmatic structures - possibly highly modified appendages - found on the underside of the opisthosoma in Amblypygi, Palpigradi and Trigonotarbida. Their function is unclear, but there have been suggestions that they play a role in osmoregulation. We score them simply as absent or present here, but note that in the different groups they occur on different segments which may raise some questions about their serial homology.

90. *Pygidial defensive secretions* (0 = absent; 1 = present).

Thelyphonida have long been known to use defensive secretions of acetic / caprylic acid, leading to the group’s common name ‘vinegaroon’. These secretions are derived from pygidial glands at the back of the opisthosoma. We tentatively score this as present in Schizomida too (as per Shultz 1990, character 46); although we caution that it has only be confirmed in a handful of schizomid species; see e.g. Reddell & Cokendolpher (1995).

91. *Genital acetabula* (0 = absent; 1 = present)

These structures are a characteristic trait of Acariformes among the mites, which usually bear three pairs (but see character 91) probably associated with opisthosomal segments 3–5. It is well-established that they are serially homologous with the Claparède organs or ‘Urstigmata’, sharing the same ultrastructure (Alberti 1977) and function (Bartsch 1973). They are highly likely to be appendage derivatives, judging from evidence concerning the development of the Claparède organs (Thomas & Telford 1999).

92. *Number of genital acetabula* (0 = three; 1 = two; 2 = several)

Among Acariformes the number of genital acetabula may vary in relation to their function in water and ion balance. Freshwater species in particular have an increased number of genital acetabula designed for supplying ions to the hemolymph. Only applicable to acariform mites possessing genital acetabula.

93. *Dorsal anal operculum* (0 = absent; 1 = present)
In Phalangiotarbida there is a round plate located dorsally at the posterior end of the opisthosoma. This has been interpreted as the anal operculum, albeit with some reservations regarding its functional morphology. Nevertheless in well-preserved phalangiotarbids this plate always preserves dorsally and does not seem to be, for example, a ventral structure pushed up through in compression fossils. A dorsal anal operculum would be unique to phalangiotarbids.

### Sensory systems

94. *Lateral eyes* (0 = absent; 1 = present)

Lateral eyes are absent in Opiliones - except for members of the suborders Cyphophthalmi and the extinct Tetropthalmi (Garwood et al. 2014) - and Pycnogonida.

95. *Lateral eye lenses* (0 = compound; 1 = five or more pairs of lenses; 2 = three primary pairs [excluding any microlenses]; 3 = two pairs; 4 = one pair)

The eyes of most of Arachnomorpha are compound in nature, as are the eyes of modern Xiphosura. This was apparently also the case in many Palaeozoic Scorpiones, Eurypterida and Chasmataspidida; although individual facets cannot always be resolved in the fossils. Coding otherwise follows Shultz (2007a, character 140) for arachnids. Extant Ricinulei have lateral light sensitive areas without lenses, thus they are coded as having lateral eyes but with an undetermined lens number. The same approach is adopted for rhagiidids among Acariformes mites. Inapplicable in those lacking lateral eyes.

96. *Lateral eye rhabdomes* (0 = net-like; 1 = star-shaped).

The rhabdomes of Xiphosura and Scorpiones share a distinct, star-like shape, whereas those of the remaining arachnids have a net-like arrangement. This star-shape was treated as an argument for a basal position for scorpions by, e.g., Weygoldt & Paulus (1979, character 21). The character is inapplicable to taxa lacking lateral eyes.

97. *Median eyes* (0 = four; 1 = two or three; 2 = absent).

Pycnogonida have an ocular tubercle with four eyes and it has been argued (Weygoldt & Paulus 1979) that this is plesiomorphic. All Euchelicerata have either two eyes, or have reduced them completely as in Ricinulei, Pseudoscorpiones, and Schizomida; see e.g. Weygoldt and Paulus (1979, character 14), Giribet et al. (2002, character 1). They can be either present or absent among Acariformes although they are absent in all Anactinotrichida.

98. *Retinula cells of medial eyes* (0 = organized into closed rhabdomes; 1 = organized into a network of rhabdomeres; 2 = disorganized; 3, inverse retina; - = inapplicable due absence of median eyes)

Scoring follows Shultz’s (2007a) character 137.

99. *Slit sense organs* (0 = absent; 1 = present).

These slit-shaped structures function as cuticular strain-gauges. They can be grouped together into so-called lyriform organs in some taxa and are often referred to as lyrifissures in mites. Slit sense organs have been recorded in all Arachnida *except* Palpigradi; see e.g. Shultz (1990, character 47) and Shultz (2007a, character 142). They could thus be considered a potential arachnid synapomorphy with a presumptive reversal in palpigrades.

100. *Trichobothria* (0 = absent; 1 = present).

These sensory hairs set into a specific, cup-shaped socket (the bothridium) detect air vibrations and are a key sensory system in many arachnids for detecting prey or other sources of movement in their vicinity. They are not seen in Pycnogonida and Xiphosura, but apparently occur in all arachnid orders with the exception of Solifugae, Ricinulei and Opiliones (Selden, Shear & Bonamo 1991), and probably Trigonotarbida too.

101. *Tibial trichobothria with 2-1-1-1 distribution* (0 = absent; 1 = present).

This specific pattern of trichobothria on the tibia was proposed by Shultz (1990, character 48) as an explicit synapomorphy of (Thelyphonida + Schizomida). It is inapplicable for taxa which lack trichobothria (see previous character).

102. *Prodorsal trichobothria* (0 = absent; 1 = present)

These specific trichobothria on the prodorsum are a textbook synapomorphy for acariform mites as compared to anactinotrichid species; although they are secondarily lost in several acariform taxa. As with some other ‘mite-specific’ characters, they can be difficult to assess in (the usually much larger) non-acarine taxa.

103. *Pectines* (0 = absent; 1 = present).

These unique structures in Scorpiones appear to be modified appendages, which primarily act as chemosensory organs. Although occurring immediately behind the gonopore, authors such as Weygoldt & Paulus (1979) argued that they belonged to the 2^nd^ (or genital) segment as part of a 12-segmented groundplan for the opisthosoma. Hox gene data from Simonnet, Célérier & Quéinnec (2006) corroborated by morphological data from Shultz (2007b) indicates that the pectines are in fact derived from the 3^rd^ opisthosomal segment as part of a 13-segmented opisthosoma. It is worth noting that recent studies of some early fossil scorpions failed to find pectines, even in well otherwise ventrally well-preserved material - for example see *Proscorpius* (Dunlop, Tetlie & Prendini 2008) and *Compsoscorpius* (Legg et al. 2012). It is conceivable that they were genuinely absent in the most basal stem-group scorpions which (if true) would render them no longer synapomorphic for the whole Scorpiones clade. The coding of *Palaeoscorpius* reflects the uncertainty reported by Kühl et al. (2012). See also the section on respiratory organs for an account of the alignment of respiratory organs on the euchelicerate opisthosoma.

104. *Malleoli* (0 = absent; 1 = present).

These unique sensory structures, sometimes called racquet organs, are a convincing autapomorphy for Solifugae, where they occur on the basal articles of the posterior legs (Punzo 1998).

105. *Tarsal organ on leg I* (0 = absent; 1 = present).

The tarsal organs or Haller’s organs occurs on leg I of Opilioacariformes, Holothryda and Ixodida (Klompen 2000), in leg I and II of Ricinulei (Talarico et al. 2005) and in all legs of Araneae and, perhaps, Scorpiones (Foelix 1985). Scoring of this – and the subsequent character – follows Shultz (2007a, characters 149, 150).

106. *Tarsal organ on leg II* (0 = absent; 1 = present).

A tarsal organ, similar to the character above, is present in Araneae and Ricinulei leg II.

107. *Opisthosomal ganglia in adults* (0 = absent; 1 = present).

In some arachnids, such as most spiders and all mites, there is a tendency to consolidate the ganglia of the central nervous system (CNS) anteriorly into the prosoma, effectively forming a unitary ‘brain’. In other taxa ganglia remain along the length of the CNS into the opisthosoma; the plesiomorphic condition on the basis of outgroup *Alalcomenaeus cambricus* (Tanaka et al. 2013).

108. *Perineural membrane enveloping arterial sinus* (0= present; 1 = absent).

Firstman (1973) related this membrane structure to the presence of book lungs and it thus scores as present for all (living) lung-bearing taxa.

109. *Intercheliceral median organ* (0 = absent; 1= present).

This tiny movable structure emerges from beneath the prosomal shield and between the chelicerae of Palpigradi. Whether it represents a modified seta or, conceivably, a vestigial element homologous with some sort of precheliceral appendage is unclear. Van der Hammen (1982) speculated if it could be homologous with the Acariformes naso.

### Respiratory system

110. *Respiratory organs* (0 = book gills or lungs present; 1 = tracheae; 2 = absent).

In this character, no attempt is made of distinguishing between lamellate gills and lungs. Their differences, as highlighted by Scholtz & Kamenz (2006) are recognized and coded separately below. Although traditionally scored as a ‘lungs absent/present’ character, our revised coding recognises that the ground pattern in the aquatic common ancestor was presumably gills, retained today in Xiphosura. In (modern) Scorpiones and Tetrapulmonata these have been transformed into lungs. Note that many derived spiders have both lungs *and* trachea (hence the 0/1 score) while various chelicerates respire only with trachea, have lost the respiratory organs all together, or perhaps never developed such structures at all (Palpigradi, Pycnogonida, some mites?). A simple division into pulmonate and apulmonate arachnids has been criticized in the past and we score this character with reservations given that not all lungs and/or trachea in arachnids open in serially homologous positions. Some of these difficulties are reflected in the characters elaborated below.

111. *Book lung/gill on 2^nd^ (i.e. genital) opisthosomal segment* (0 = present; 1 = absent).

Assuming the plesiomorphic condition was a series of respiratory organs along the trunk/opisthosoma, it is noticeable that in some chelicerates these have been lost on particular segments. As putative basal euchelicerates, *Offacolus* and *Dibasterium* bear a series of flattened appendages along the opisthosoma from the second to the seventh segment, the first to third among them bearing preserved accessory flaps in the former (Sutton et al. 2002), and first to fourth in the latter (Briggs et al. 2012). A similar state is observed in *Weinbergina*. By contrast Xiphosura and Scorpiones are notable for completely lacking a lamellate respiratory organ on the second (or genital) opisthosomal segment.

112. *Book lung/gill on 3^rd^ (i.e. postgenital) opisthosomal segment* (0 = present; 1 = absent).

A lamellate respiratory organ has been retained on this segment in: both modern Xiphosura and the fossil horseshoe crabs *Weinbergina*, *Dibasterium*, and *Offacolus*; in Trigonotarbida; and in Tetrapulmonata. They have been lost in segment 3 in Scorpiones – whereby it is interesting to speculate whether the pectines in this position are homologous appendicular elements – and, perhaps, in Eurypterida too (Braddy et al. 1999). Note that in more derived spiders (Araneae: Araneoclada) the second book lung has almost certainly been modified directly into trachea. This condition is also scored 1 here, but is presumably homoplastic with respect to other euchelicerates.

113. *Book lung/gill on 4^th^ to 7^th^ opisthosomal segment* (0 = present; 1 = absent).

A lamellate respiratory organ has been retained on these segments in both modern Xiphosura and the fossil horseshoe crabs *Weinbergina*, *Dibasterium*, and *Offacolus*; and in Scorpiones. The gill/lung has been (apomorphically) lost on these segments in the Pantetrapulmonata. Note that characters 111–113 are related to the transformation of book gills into book lungs, thus they are again coded only for taxa fundamentally bearing lamellate respiratory organs.

114. *Spiracles* (0 = absent; 1 = present)

Early fossil scorpions do not express ventral spiracles opening within the relevant sclerite. It is possible that the spiracle opening in fossil scorpions was marginal on a segment and concealed beneath a sclerite, rather like in Pedipalpi, but this is difficult to assess from the available material. Marginal spiracles can be clearly seen in well preserved examples of Trigonotarbida. In the coding adopted here, it was assumed that the spiracles of Scorpions and Tetrapulmonata are homologous, a controversial assumption; see also character 117.

115. *Spines on book lung lamellar margins* (0 = absent; 1 = present)

All book lungs express spines from the margins of the lamellae pointing into the atrial chamber which possibly help filter out particles and prevent them from entering the delicate lamellae themselves (Scholtz & Kamenz 2006). These spines are absent in the xiphosuran book gills, and they can be seen in the remarkable well-preserved fossil lungs of the trigonotarbid *Palaeocharinus* (Kamenz et al. 2008).

116. *Shape of pillars of the haemolymph spaces inside the gill/lung lamellae* (0 = at least two perikarya meeting midway in the haemolymph space; 1 = pillars, including a strong axis of microtubules).

State 1 is found in Xiphosuran book gills (Scholtz & Kamenz 2006). This character is inapplicable to those taxa which lack book gills/lungs.

117. *Prosomal spiracles* (0 = absent; 1 = between the coxae of the second and third walking legs; 2 = associated with coxae of third and fourth walking leg; 3 = between cheliceral basis; 4 = brachypyline oribatid tracheal system).

Tracheal openings between coxae II and III (e.g. Giribet et al. 2002, character 25) are a potential autapomorphy of Solifugae. Openings between coxae II and IV are observed in Ricinulei and are tentatively scored here for Anactinotrichida except *Opilioacarus* (see subsequent character). Spiracular openings between the chelicerae are found in some prostigmatid mites – hence their name – although further taxon-specific respiratory structures are found among ingroup members (Evans 1992, Alberti and Coons 1999). Most Astigmata and Endeostigmata lack respiratory organs. Brachypyline oribatids have spiracles opening in acetabula or sockets of legs I and II and between legs II and III. Among Oribatida many other respiratory structures occur, but the details do not seem to be informative for a higher level phylogeny (Alberti & Coons 1999). This character touches on the question of whether tracheal systems can be easily reduced to simple presence/absence characters and/or whether the segment on which the respiratory organ opens carries as much phylogenetic information as the fact that it is a lung or a trachea; see also comments below. On current data a simple answer to this question does not present itself.

118. *Opisthosomal spiracles* (0 = absent; 1 = paired ventral stigmata on genital segment; 2 = paired ventral stigmata on 3^rd^ and 4^th^ opisthosomal segments; 3 = four pairs of dorsal stigmata on the anterior opisthosoma).

Character state 1 is scored for Opiliones. State 2 is scored for Pseudoscorpiones and Solifugae, although the latter also has an unpaired spiracle on the 5^th^ opisthosomal segment. State 3 is autapomorphic for Opilioacariformes. If tracheae are derived from book-lungs it is likely that the positions of the tracheal openings are serially homologous with the relevant book lungs in other arachnids. In the absence of unequivocal lung/trachea homology across all arachnids (cf. the prosomal or dorsal opisthosomal spiracles above) we score these here as independent characters for now.

119. *Kiemenplatten* (0 = absent; 1 = present)

Well-preserved eurypterids uniquely possess modified oval areas on the underside of the body, located within the gill chambers. These are conventionally referred to as Kiemenplatten or sometimes simply ‘gill tracts’. Assuming that eurypterids retained lamellate book gills, these Kiemenplatten have been interpreted as an accessory respiratory system with possible parallels to the branchial lungs of certain modern crabs which allow these animals to undertake temporary excursions onto land.

### Digestive system

120. *Postcerebral crop and proventriculus* (0 = reduced, 1 = present).

A large crop is associated with the posteriorly-directed mouth in Xiphosura. They may thus form part of a character complex together. Such a crop is not recorded in arachnids or pycnogonids and reduction of the crop was proposed as a putative synapomorphy of Arachnida by Shultz (2007a, character 202).

121. *Well-developed* s*ucking stomach* (0 = absent; 1 = present).

Clearly present in Araneae and Amblypygi, and treated as a synapomorphy of these taxa by e.g. Weygoldt & Paulus (1979, character 31). Shultz (1990, character 5) noted its vestigial presence in Uropygi and some Scorpiones too, while Shultz (2001) further noted that Xiphosura also have a muscular postcerebral pharynx. Scoring follows Weygoldt & Paulus (1979).

### Endosternite

122. *Endosternite* (0 = absent; 1 = present).

This structure may be homologous with Dohrn’s septum in Pycnogonida, but it occurs in a specifically plate-like form in Euchelicerata; with the exception of Solifugae. Firstman (1973) gave a detailed account, whereby its absence in Solifugae was considered a secondary reversal. Shultz (1990) also sought to define an endosternite *sensu stricto* as “a broad sheet of non-contractile connective tissue”.

123. *Anterior endosternal horn* (0 = terminating in muscular attachment to labrum; 1 = terminating in muscular attachment to palpal coxa).

This specific morphology of the endosternal horn was proposed by Shultz (1990, character 4) as a putative synapomorphy of Pedipalpi. It is inapplicable to taxa which lack an endosternite (see above).

124. *Fenestrate endosternite* (0 = absent; 1 = present).

This specific form of the endosternite is restricted to Thelyphonida and Schizomida (1990, character 8). It is inapplicable to taxa which lack an endosternite (see above).

### Excretory organs

125. *Malpighian tubules* (0 = absent; 1 = present).

These excretory organs are another ‘typical’ textbook arachnid character. They have been recorded in all Recent arachnids *except* Palpigradi, Opiliones, Pseudoscorpiones and most Acariformes; see e.g. Shultz (1990, character 62). Among Acariformes, structures similar to Malpighian tubules have been found in Acaridae, thus they are tentatively scored here as present.

126. *Coxal glands opening on proximal podomere of chelifore* (0 = absent; 1 = present).

Coxal glands are modified nephridia and as such of mesodermal origin. The coxal gland opening is an ectodermal invagination and may not correspond to the segment from which the coxal gland sacculi originate. We prefer a simplified account for the coxal glands considering an anterior and a posterior coxal gland opening as the Bäuplan condition, as has been suggested by Weygoldt (2000). It reflects our poor understanding of the embryology and organogenesis of several orders. Fahrenbach & Arango (2007) described the presence of a pair of coxal glands associated with the *Nymphopsis* *spinosissima* chelifores – the first occurrence of excretory organs of any kind in Pycnogonida. King (1973) suggested that excretory organs may be present on other leg bases based on dying techniques, but these results require confirmation. We accept here the existence of this anterior pair of coxal glands, but current data does not rule out the existence of more posterior pairs. Therefore, the following two characters are scored as ambiguous for Pycnogonida.

127. *Coxal glands opening at base of leg 1* (0 = absent; 1 = present).

State 0 is coded here for all living Euchelicerata, except Xiphosurans, Scorpiones, Opiliones and Pseudoscorpiones. Coxal gland openings in this position are thus retained in Acari, Ricinulei, Palpigradi, Solifugae, Araneae, Amblypygi, Thelyphonida and Schizomida (e.g. Shultz 1990, character 64; Shultz 2007a, character 180). Opening of the coxal glands associated with the pedipalps (the second prosomal segment) have been mooted as an autapomorphy of Solifugae (Shultz 1990), but Buxton (1917) described them opening in the same position in Palpigradi. The condition of Solifugae is quite similar to that found in several Acariformes were a cuticle-lined channel lead the fluids of the coxal gland towards the pre-oral chamber. It is worth also mentioning the so-called ‘hatching glands’ associated with the pedipalp in spider embryos (Yoshikura 1975) as well the second ozopore in some opilionids (Hara 2003) could be homologous with coxal glands (Moritz 1959, Yoshikura 1975).

128. *Coxal glands opening on leg 3 segment* (0 = absent; 1 = present).

Coxal gland openings in this position are plesiomorphically retained in Xiphosura, mygalomorph Araneae, basal Amblypygi, Opiliones, Scorpiones and Pseudoscorpiones (e.g. Shultz 1990, character 63). It is scored as ambiguous for the extinct Eurypterida except *Baltoeurypterus* (now *Eurypterus*) where Selden (Selden 1981) reported a pit near insertion of leg III.

129. *Contribution of the coxal gland to saliva* (0 = absent; 1 = Buxton’s group II coxal gland; 2 = coxal glands and saliva converging into the pre-oral chamber through external taenidia or gutters; 3 = podocephalic channel).

The onycophoran salivary gland is a modified nephridium, where a terminal sac lined with podocites is found along with a hypertrophied secretory region (Buxton 1913; Storch, Alberti & Ruhberg 1979). This structure is quite similar to the coxal glands of Palpigradi and Solifugae (Buxton 1913, 1917; Alberti 1979). In Solifugae the putative function of the coxal gland secretion as saliva is congruent with its opening as an excretory organ in close association to the pre-oral chamber. In Palpigradi this relation is not so clear; our coding recognizes the internal structural similarity with Solifugae (*contra* Shultz, 2007a). The condition presented by Parasitiformes approaches that of Solifugae. The so-called podocephalic channel, which leads the coxal gland secretions to the exterior, receives the products from up to three salivary glands and delivers them to the pre-oral chamber (Alberti & Coons 1999). However, each gland has its own distal portion lined by cuticle, being therefore the connection among the podocephalic glands made by an ectodermic structure (e.g. Shatrov 2005). Although functionally very similar, the condition presented by Acariformes apparently evolved from structures of different embryological origins and is coded here as a distinct state. Tetrapulmonata, Ricinulei, Holothyrida, and Opiliocariformes present prosomal furrows that lead the product from the coxal glands to the pre-oral chamber where it contributes to the saliva (Shultz 2007a, character 14). Scorpiones, Opiliones, Pseudoscorpiones, Xiphosura, Pycnogonida and ticks have no documented relationship between coxal glands and saliva secretion and are scored as 0.

130. *Dorsomedian excretory organ* (0, absent; 1, present).

This is a specialized, post-colon, region of the midgut modified for excretion which is present in Prostigmata (Alberti & Coons 1999) among the mites.

### Musculature

*Pharyngeal musculature*

131. *Lateral extrinsic precerebral pharyngeal muscle* (0 = arising from anterior endosternal horns; 1 = arising from medial surface of palpal coxae; - inapplicable for taxa lacking an endosternite).

This specific arrangement of the pharyngeal musculature was proposed by Shultz (1990, character 6) as a putative synapomorphy of Pedipalpi.

132. *Ventral extrinsic precerebral pharyngeal muscle* and *tergopharyngeal muscle of precerebral pharynx* (0 = present; 1 = absent).

A further specific arrangement of the pharyngeal musculature was proposed by Shultz (1990, character 7 and 8) as a putative synapomorphy of Pedipalpi. Shultz (1990) interpreted these two muscles listed above as independent characters, but we prefer to treat them as a single character complex in order to avoid weighting the analysis too heavily towards patterns of individual muscle insertions.

*Cheliceral musculature*

133. *Cheliceral tergal–deutomerite muscle* (0 = absent; 1 = present).

Among those taxa whose chelicerae have three articles, this muscle running from the prosomal shield to the proximal margin of the second article (the deutomerite *sensu* Shultz) has so far only been recorded in Scorpiones and Opiliones (Shultz 2000), for which it was proposed as a putative synapomorphy. It is scored as inapplicable here for taxa with only two cheliceral articles.

134. *Lateral tergocheliceral muscle* (0 = one head; 1 = three heads).

This specific pattern of tergocheliceral musculature was proposed by Shultz (1990, character 10) as a putative synapomorphy of Pedipalpi.

135. *Paired muscle arising from posterior margin of anterior carapacal doublure and inserting on prosomal shield*  (0 = absent; 1 = present).

Shultz (1990, character 1) described this specific muscle pairing as a putative synapomorphy of Pedipalpi.

*Endosternal musculature*

136. *Posterior oblique muscles of box*–*truss axial muscle system (BTAMS) of postoral somites I–*V (0 = absent; 1 = present in one or more somites).

Shultz (2001) investigated the so-called box–truss axial muscle system in detail. State 0 occurs in Xiphosura, and state 1 occurs in Palpigradi, Araneae, Amblypygi, Thelyphonida, Schizomida and Scorpiones (Shultz 2001; 2007a, character 127). The condition in Acari, Ricinulei, Opiliones and Solifugae is not recorded.

137. *Anterior oblique muscles of BTAMS posterior to postoral somite VI* (0 = absent; 1 = present).

State 1 occurs in Xiphosura and may represent the primitive condition based on comparison with other arthropods (Shultz 2001; 2007a, character 128). State 0 occurs in all arachnids examined thus far.

138. *Intercoxal endosternal extensor muscles* (0 = absent; 1 = present).

The presence of these specific muscles was tentatively proposed as a synapomorphy of (Scorpiones + Opiliones) by Shultz (2000).

139. *Endosternal dorsal suspensors of somites I and II* (0 = present; 1 = absent/detached).

The absence of these suspensor muscles was suggested by Shultz (1990) as a putative synapomorphy of Arachnida; although the possibility that these muscles have become modified in other ways was also discussed; see Shultz (2001, p. 301) for details. Inapplicable to taxa which lack an endosternite (see above).

140. *Endosternal dorsal suspensor muscles in somite four with anterolateral carapacal insertion* (0 = absent; 1 = present).

The specific insertion of the endosternal suspensor muscles in Palpigradi, basal Araneae, Amblypygi and Uropygi is more posteromedial and often associated with the median prosomal shield depression typically seen in such arachnids (Shultz 1990, character 7). Inapplicable to taxa which lack an endosternite (see above).

141. *Endosternal dorsal suspensor muscle of somite five* (0 = present; 1 = absent).

This specific pattern of endosternal musculature was proposed by Shultz (1990) as a putative synapomorphy of Pedipalpi. Inapplicable to taxa which lack an endosternite (see above).

142. *Ventral endosternal suspensor muscles* (0 = attaching primarily to sternum; 1 = attaching primarily to coxa of appendage of anteriorly adjacent somite).

This specific pattern of musculature was proposed by Shultz (1990, character 7) as a putative synapomorphy of Pedipalpi. Inapplicable to taxa which lack an endosternite (see above).

*Pedipalp musculature*

143. *Palpal posteromedial tergocoxal muscle* (0 = present; 1 = absent).

This specific pattern of tergocoxal musculature was proposed by Shultz (1990) as a putative synapomorphy of Pedipalpi.

144. *Palpal posteromedial endosternocoxal muscle* (0 = originates on endosternite, inserts on coxa; 1 = originates *and* inserts on coxa).

This specific pattern of endosternal musculature was proposed by Shultz (1990) as a putative synapomorphy of Pedipalpi.

*Leg musculature*

145. *Intracoxal muscle* (0 = absent; 1 = present).

This specific muscle in the walking leg coxae was proposed by Shultz (2007a, character 54) as a putative synapomorphy of Pedipalpi.

146. *Insertion process of anteromedial tergocoxal muscle* (0 = weakly developed; 1 = large, well developed).

This specific pattern of leg musculature was proposed by Shultz (2007a, character 57) as a putative synapomorphy of Pedipalpi.

147. *Femoropatellar flexor* (0 = symmetrical; 1 = asymmetrical).

The asymmetrical arrangement of this muscle complex in the leg was proposed by Shultz (1990, character 27) as a putative synapomorphy of Pedipalpi.

148. *Pedal anterior femur–patella muscle* (0 = inserting primarily on patellar margin; 1 = inserting primarily on patellar plagula).

This specific pattern of leg musculature was proposed by Shultz (1990, character 27) as a putative synapomorphy of Pedipalpi.

149. *Pedal posterior femeropatella-tibia muscle* (0 = present; 1 = absent).

Absence of this specific muscle in the walking legs was proposed by Shultz (1990, character 33) as a putative synapomorphy of Pedipalpi.

150. *Pedal patellotibia-tarsus muscle* (0 = present; 1 = absent).

Absence of this specific muscle in the walking legs was proposed by Shultz (19, character 29) as a putative synapomorphy of Pedipalpi.

151. *Posterior transpatellar muscles insertion* (0 = dorsoposterior femur/ posterior patella; 1 = distal process of femur; 2 = absent).

Shultz (1990 character 28) regarded a femoral and/or patellar origin of this muscle as the plesiomorphic condition, regarding its specific origin from a distodorsal process as an apomorphy seen in Scorpiones, Opiliones and Pseudoscorpiones. This muscle is absent in Solifugae, Ricinulei and Schizomida, which is interpreted as a further derived state.

152. *Patellotibial extensor* (0 = absent; 1 = present).

According to Shultz (1990, character 29) the posterior transpatellar muscle in Scorpiones and Pseudoscorpiones has a specific dorsal insertion point and appears to act as a patellotibial extensor.

153. *Anterior transpatellar muscle insertion* (0 = anteriorly/anteroventrally; 1 = ventrally / posteroventrally; 2 = absent).

This muscle was reported (1990, character 30) as normally inserting on the anterior margin of the tibia, but as inserting ventrally in Scorpiones, Pseudoscorpions and Solifugae and as being absent in Ricinulei and Schizomida.

154. *Anterior patellotibial muscle insertion on tibia* (0 = anterior; 1 = ventral; 2 = absent).

Shultz (1990, character 32) described this muscle as having a ventral insertion point in Ricinulei, Scorpiones and Solifugae and as (uniquely) being absent in Pseudoscorpiones.

155. *Posterior patellotibial muscle* (0 = present; 1 = absent).

This specific muscle was reported by Shultz (1990, character 33) as apomorphically absent in Scorpiones, Pseudoscorpiones, Solifugae and Schizomida.

156. *Origin of apotele depressor* (0 = tarsus; 1 = tibia).

A tibial origin of the apotele (or pretarsus) depressor muscle was suggested by Shultz (1990, character 35) as a possible synapomorphy for Arachnida.

157. *Patellar head of apotele depressor* (0 = absent; 1 = present).

The apotele depressor muscle has a head extending into the patella in Araneae, Thelyphonida, Schizomida, Opiliones, Scorpiones, Pseudoscorpiones and Solifugae Shultz (1989, 1990 character 36).

158. *Patellar head of apotele depressor originates on posterior patellar wall* (0 = absent; 1 = present).

This specific position of the patella head of these depressor muscles was suggested by Shultz (1990, character 37) as a putative synapomorphy for Thelyphonida and Schizomida. It is not applicable to those taxa (see above) that lack a patella head.

*Opisthosomal musculature*

159. *Attachments of opisthosomal posterior oblique axial muscles*. (0 = tergal; 1 = pleural).

The pleural attachment of these muscles was suggested as a possible synapomorphy for Arachnida by Shultz (2001). Given the highly reduced trunk in extant forms, all these opisthosomal musculature characters are scored here as inapplicable for Pycnogonida.

160. *Opisthosomal pleural muscle* (0 = continuous dorsoventral sheet; 1 = divided into three components).

Division of this opisthosomal muscle sheet was proposed by Shultz (1999, character 28) as a putative synapomorphy of Pedipalpi.

161. *Dorsal and ventral longitudinal muscles* (0 = spanning full length of opisthosoma; 1 = spanning first and, perhaps, last four opisthosomal somites).

This specific pattern of longitudinal muscles was proposed by Shultz (1999, character 30) as a putative synapomorphy of Pedipalpi. How this should apply to acariform mites with their rather short ‘opisthosoma’ is unclear and they have been scored (?) here.

### Reproduction

162. *Internal fertilisation* (0 = absent; 1 = present).

Male xiphosurans release sperm directly onto the eggs and thus fertilise them externally (Aberti 2000) – presumably the plesiomorphic behaviour – whereas all arachnids use various mechanisms (outlined as individual characters below) to achieve internal fertilisation. This is a putative synapomorphy of Arachnida. The precise mode of sperm transfer in Pycnogonida is uncertain (Alberti 2000), as is that of various fossil taxa. There is some circumstantial evidence that the extinct eurypterids used internal fertilization too with sperm perhaps being deposited and/or taken up by the genital appendage.

163. *Gonopores* (0 = on limb bases; 1 = on second opisthosomal segment).

The genital opening occurs on the second opisthosomal segment in all extant Euchelicerata, whereas Pycnogonida have genital openings on the limb bases. Whether the pycnogonid condition is plesiomorphic or apomorphic is subject to debate (Dunlop & Arango 2005); arguments for both hypotheses can be formulated, such as the displacement of organ systems into the pycnogonid legs.

164. *Gonopores* (0 = paired on second opisthosomal segment; 1 = unpaired on second opisthosomal segment).

Consolidation of paired gonopores – as in for example xiphosurans – into a single genital opening has been suggested as a synapomorphy of Arachnida (Shultz 2001). The state of this character in fossil taxa, such as the eurypterids with their median abdominal appendage, is difficult to determine and scored as uncertain.

165. *Anteriorly positioned gonopore* (0 = absent; 1 = present).

In Opiliones and Ixodida, the genital opening is thrust forwards into a distinctly anterior position more or less between the leg coxae; see e.g. Giribet et al. (2002, character 165).

166. *Ovipositor* (0 = absent; 1 = present).

An ovipositor is present in Opiliones and in *some* Anactinotrichida – with the important exception of the ticks – as well as Acariformes among the mites sampled here.

167. *Penis / Spermatopositor* (0 = absent; 1= present; 2= acariforme aedagus).

A penis is unequivocally present in Opiliones. A penis-like structure is also seen in *some* acariform mites; however in the latter group it is probably better to regard it as a spermatopositor which has (homoplastically) evolved into a copulatory device in various ingroup mites. Thus a true penis, as per Opiliones, may not be part of the acariform mite ground pattern. Anactinotrichid mites do not have a penis at all and attempts to use a penis to support (Opiliones + all Acari) are misleading. Indeed among Acariformes the presence of a “penis” has probably evolved several times. All are scored as “2” here, however, in Astigmata, Cheyletidae, Tetranychoidea and Stigmaeidae.

168. *Male palpal organ* (0 = absent; 1 = present).

Direct sperm transfer via the modified palpal organ of mature male spiders is an autapomorphy of Araneae. The uraraneid palp is unknown.

169. *Sperm transfer device on leg 3* (0 = absent; 1 = present).

Functionally similar to the spider palpal organ (see above), a modified organ for direct sperm transfer on the third leg in mature males is an unequivocal autapomorphy of Ricinulei.

170. *Stalked spermatophore* (0 = absent; 1 = present).

Alberti (2000) commented on the possibility that some sort of spermatophore was part of the arachnid ground pattern, but noted difficulties in defining what exactly constitutes a spermatophore *sensu stricto*. Scorpiones, Pseudoscorpiones, Amblypygi, Thelyphonida and Schizomida transfer sperm via an explicitly stalked spermatophore; e.g. Shultz (1990, character 57). Among mites most of Acariformes produces spermatophores while most of Anactinothrichida exhibits espermdactly insemination or transfer the sperm employing the chelicerae. The exact mechanism of sperm transfer in Opilioacariformes and Holothyrida remains unknown.

171. *Spermatophore uptake* (0 = without mating; 1 = face-to-face uptake; 2 = mating parade).

Various arachnids perform ritualised mating behaviours or ‘dances’. In Scorpiones and Amblypygi the male manoeuvres the female over the spermataphore with the animals face-to-face. This is often called the ‘promenade-de-deux’ in scorpions and this face-to-face behaviour is tentatively treated as a potential apomorphy, although details of courtship may differ between groups. Pseudoscorpiones are more complex. Some, probably basal, taxa simply leave a spermataphore and do not strictly speaking mate (Weygoldt 1969; Alberti 2000). Others have a scorpion-like courtship. We score pseudoscorpions 0/1 here for the character. A specific behaviour pattern identified for Thelyphonida and Schizomida is what Weygoldt (1978) termed the ‘mating parade’ (see also Shultz 1990; character 58) in which the female grabs the male opisthosoma and is pulled over a previously deposited spermatophore with the animals facing in the same direction. This character is inapplicable for taxa which do not use spermatophores and the behaviour of Acariformes is too diverse to allow useful coding of this character at this stage.

172. *Testis* (0 = glandular area unspecialised; 1 = glandular area distinctly larger).

Alberti & Peretti (2002) described distinct and potentially apomorphic similarities in the morphology of the testis in Solifugae and Acariformes only among the mites. In detail, they observed that in both groups there is a large glandular area, probably producing secretions needed for spermatophore formation, which has not been observed in other arachnids.

173. *Tubular genital accessory glands* (0 = absent; 1 = present).

These so-called holocrine glands occur among arachnids only within the genital system of Amblypygi, Thelyphonida and Schizomida (Alberti 2000, 2005), and thus potentially support the Pedipalpi clade.

174. *Brood sac* (0 = absent; 1 = present).

Pseudoscorpiones, Amblypygi, Thelyphonida and Schizomida construct a brood sac in which eggs – or in the case of pseudoscorpions, embryos – develop; see e.g. Shultz (1990, character 59) and Shultz (2007a, character 172).

### Sperm morphology

175. *Sperm cell flagellum* (0 = present; 1 = absent).

Plesiotypic sperm cells are supposed to be flagellate among animals. Loss of the flagellum is associated with specialization of the sperm transfer mechanisms.

176. *Sperm cells coiled* (0 = present; 1 = absent).

This character is inapplicable for taxa without a flagellum.

177. *Microtubule arrangement in axoneme* (0, 9 + 0; 1, 9 + 1; 2, 9 + 2; 3, 9 + 3 )

The 9x2 + 3 arrangement of microtubules in the sperm axoneme is widely regarded as a convincing synapomorphy of Tetrapulmonata (Alberti 2000, 2005 and references therein). Note that some ingroup spiders show further modifications of this pattern (Michalik & Alberti 2005). Inapplicable in taxa without an axonem.

178. *Corkscrew-shaped helical nucleus* (0 = absent; 1 = present).

The specific morphology of a helically-shaped nucleus with sharp edges was suggested by Alberti (2000, 2005) as a potential synapomorphy of (Araneae + Amblypygi). Pseudoscorpiones were also noted as having a corkscrew-shaped nucleus, but here derived from a peculiar spiral band. This raises questions about whether it is really homologous with the spider/whip-spider condition and pseudoscorpions are thus scored (?) here for this character.

179. *Postcentriolar nucleus* (0 = unmodified; 1 = asymmetrical, elongate).

The latter condition of the nucleus was suggested as a potential synapomorphy for (Araneae + Amblypygi) by Alberti (2000, 2005).

180. *Implantation fossa* (0 = shallow; 1 = deep).

In arachnids with flagellate sperm the implantation fossa – a posterior part of the nucleus which usually contains the centrioles or their derivatives (Alberti 2000) – is usually shallow, but in many Araneae and Schizomida it is deep and effectively makes the sperm an almost a hollow tube. This character is inapplicable to aflagellate sperm, and only visible in basal Opiliones which develop a transient flagellum.

181. *Manchette of microtubules* (0 = absent; 1 = present).

Alberti (2000) noted that a manchette of microtubules associated with the nucleus – and perhaps related to nuclear shaping – is only seen in Ricinulei and the Tetrapulmonata orders; see also Giribet *et al*. (2002).

182. *Nuclear envelope* (0 = absent; 1 = present).

A nuclear envelope, which disappears at the end of spermatogenesis, is observed in Solifugae and Acariformes among the mites, although a similar phenomenon may occur in some Xiphosuran (Alberti 1995, 2000).

183. *Persisting flagellar tunnel* (0 = absent; 1 = present).

This tunnel surrounding the axoneme persists throughout spermiogenesis only in Scorpiones and Pseudoscorpiones (Alberti 2000); see also Giribet et al. (2002, character 203).

184. *Vacuolated sperm* (0 = absent; 1 = present).

Alberti (2000, especially fig. 33) described the quite fundamental differences between the sperm types seen in the Anactinotrichida and Actinotrichida groups of mites. He thus concluded that at least the sperm characters offer no characters in support of monophyletic Acari. Vacuolated sperm were suggested as a synapomorphy of Anactinotrichida, with some further modifications to this ground pattern to form ‘ribbon-type’ sperm in the gamasid mites.

### Embryology/Development

185. *Eggs* (0 = centrolecithal; 1= isolecithal/telolecithal).

Yohsikura (1975, table 1) described the eggs of most arachnids as centrolecithal. The eggs of scorpions and pseudoscorpions are quite different and show yolk reduction. Yoshikura (1975) related this reduction to ovoviviparity and viviparty in scorpions and the laying of eggs in a brood pouch in pseudoscorpions, both of which consequently reduce the reliance on yolk.

186. *Growth zone* (0 = gives rise to prosoma *and* opisthosoma; 1 = gives rise to opisthosoma only).

Anderson (1973) and Yoshikura (1975) described a fairly fundamental difference in the way the opisthosoma develops in Xiphosura and Scorpiones, as compared to non-scorpion arachnids. In the former, the growth zone gives rise to both tagmata, in the latter the prosoma develops directly from the blastoderm (see also Giribet et al. 2002, character 192).

187. *Hexapodal instar* (0 = absent; 1 = present).

An early instar with only six legs is one of the strongest character proposed in support of (Ricinulei + Acari) – see e.g. Lindquist (1984), and Shultz (1990, character 61) – although it should be cautioned that adding limbs during ontogeny *could* be treated as a plesiomorphic, anamorphic mode of development.

188. *Egg teeth on pedipalpal coxae* (0 = absent; 1 = present).

Yoshikura (1975) observed various serrations in arachnids which probably function to break open the egg while hatching. Of these, a specific pair of teeth restricted to the dorsal surface of the pedipalpal coxae, and which are shed after hatching, was mentioned for Araneae and Amblypygi and is thus a potential synapomorphy of these taxa (see also Wheeler & Hayashi 1998, character 32). It is not clear to what extent this character has been investigated in other taxa.

189. *Lateral organs* (0 = absent; 1 = present).

This is a problematic character, in that the lateral organs seen during development in Solifugae, Amblypygi and Uropygi (Yoshikura 1975) may well be vestigial, plesiomorphic retentions of an exopod from the base of the second walking leg and are probably homologous with the Claperède organ of Acariformes (Thomas & Telford 1999). All four taxa are scored as having this character here. There is a so-called lateral organ in Xiphosura too, but Thomas & Telford (1999) questioned whether it was really homologous with that of arachnids – it does not really occur on the limb base in horseshoe crabs – and this is reflected in the scoring adopted here where its presence is restricted to the above-mentioned arachnids.

### Ecology

191. *Heteromorphic parasitic larvae* (0 = absent; 1 = present).

This specialised life cycle with an obligatory parasitic larva is a putative apomorphy of the Parasintegona group among prostigmatid mites.

192. *Hypopi* (0 = absent; 1 = present).

The deutonymphal stage in Astigmata has reduced mouthparts, provided with a posterior clasping or sucking device related to dispersion by phoresis. This trait is exclusive to Astigmata (*Sancassania* and *Rhizoglyphus* among the taxa sampled in this study) among the mites.

193. *Anamorphic development with protonymphal stage* (0 = present, 1 = absent)

Pycnogonida show anamorphic development in which the hatching stage, or protonymph, is fundamentally different from the adult in having less segments and appendages. Subsequent segments and limbs are added during development. Xiphosurans and arachnids hatch as miniature versions of the adult, although in some cases the full complement of limbs is only achieved with later instars (see hexapodal larva).

**References**

Alberti, G. (1977). Zur Feinstruktur und Funktion der Genitalnäpfe von Hydrodroma despiciens (Hydrachnellae, Acari). Zoomorphologie, 87(2), 155-164.

Alberti, G. (1979). Licht- und elektronenmikroskopische Untersuchungen an Coxaldrüsen von Walzenspinnen (Arachnida: Solifugae). Zoologischer Anzeiger, 203, 48–64.

Alberti, G. (1995). Comparative spermatology of Chelicerata: review and perspective. Advances in Spermatozoal Phylogeny and Taxonomy.(BGM Jamieson, J. Ausio & J.-L. Justine, eds.) Mémoires du Muséum National d’Histoire Naturelle, 166, 203-230.

Alberti, G. (2000). Chelicerata. Reproductive biology of invertebrates, 9(Part B), 311-388.

Alberti, G. (2005). Double spermatogenesis in Chelicerata. Journal of morphology, 266(3), 281-297.

Alberti, G. (2006). On some fundamental characteristics in acarine morphology. Atti Accad Naz Ital Entomol, 2005, 315-360.

Alberti, G., & Coons, L. B. (1999). Acari: mites. Microscopic anatomy of invertebrates, 8, 515-1265.

Alberti, G., Lipke, E., & Giribet, G. (2008). On the ultrastructure and identity of the eyes of Cyphophthalmi based on a study of *Stylocellus* sp.(Opiliones, Stylocellidae). Journal of Arachnology, 36(2), 379-387.

Alberti, G., & Peretti, A. V. (2002). Fine structure of male genital system and sperm in Solifugae does not support a sister-group relationship with Pseudoscorpiones (Arachnida). Journal of Arachnology, 30(2), 268-274.

Anderson, D. T. (1973). Embryology and phylogeny in annelids and arthropods. Pergamon Press, Oxford, UK.

Anderson, L. I., & Selden, P. A. (1997). Opisthosomal fusion and phylogeny of Palaeozoic Xiphosura. Lethaia, 30(1), 19-31.

Arango, C. P. (2002). Morphological phylogenetics of the sea spiders (Arthropoda: Pycnogonida). Organisms Diversity & Evolution, 2(2), 107-125.

Barnett, A. A., & Thomas, R. H. (2013). The expression of limb gap genes in the mite *Archegozetes longisetosus* reveals differential patterning mechanisms in chelicerates. Evolution & development, 15(4), 280-292.

Bartsch, I. (1973). *Porohalacarus alpinus* (Thor)(Halacaridae, Acari), ein morphologischer Vergleich mit marinen Halacariden nebst Bemerkungen zur Biologie dieser Art. Entomol tidskr, 74, 116-123.

Börner, C. (1902). Arachnologische Studien (II und III). Zool. Anz, 25, 433-466.

Braddy, S. J., Aldridge, R. J., Gabbott, S. E., & Theron, J. N. (1999). Lamellate book‐gills in a late Ordovician eurypterid from the Soom Shale, South Africa: support for a eurypterid‐scorpion clade. Lethaia, 32(1), 72-74.

Briggs, D. E., Siveter, D. J., Siveter, D. J., Sutton, M. D., Garwood, R. J., & Legg, D. (2012). Silurian horseshoe crab illuminates the evolution of arthropod limbs. Proceedings of the National Academy of Sciences, 109(39), 15702-15705.

Bruton, D. L. (1981). The arthropod Sidneyia inexpectans, Middle Cambrian, Burgess Shale, British Columbia. Philosophical Transactions of the Royal Society of London. Series B, Biological Sciences, 619-653.

Buxton, B. H. (1913). Coxal glands of arachnids. Zoologische Jahrbücher, Abteilung für Anatomie und Ontogenie der Tiere, 14, 231–282.

Buxton, B. H. (1917). Notes on the anatomy of arachnids. Journal of Morphology, 29(1), 1-31.

Chamberlin, J. C. (1931). The arachnid order Chelonethida. Stanford University Press Stanford USA.

Cotton, T. J., & Braddy, S. J. (2003). The phylogeny of arachnomorph arthropods and the origin of the Chelicerata. Transactions of the Royal Society of Edinburgh: Earth Sciences, 94(03), 169-193.

Damen, W. G., Saridaki, T., & Averof, M. (2002). Diverse adaptations of an ancestral gill: a common evolutionary origin for wings, breathing organs, and spinnerets. Current Biology, 12(19), 1711-1716.

Dencker, D. V. (1974). Das Skeletmuskelsystem von *Nymphon rubrum* Hodge, 1862 (Pycnogonida: Nymphonidae). Zool. Jbr. Anat, 93, 272-287.

Dunlop, J. A. (1996). Evidence for a sister group relationship between Rininulei and Trigonotarbida. Bulletin of the British Arachnological Society, 10, 193-204.

Dunlop, J. A. (1999). A redescription of the Carboniferous arachnid *Plesiosiro madeleyi* Pocock, 1911 (Arachnida: Haptopoda). Transactions of the Royal Society of Edinburgh, Earth Sciences 90,29–47.

Dunlop, J. A. (2000a). The epistomal-labral plate and lateral lips in solifuges, pseudoscorpions and mites. Ekol. Bratslava, 19, 67-78.

Dunlop, J. A. (2000b). Character states and evolution of the chelicerate claws. European arachnology 2000, 345-354.

Dunlop, J. A. (2010). Geological history and phylogeny of Chelicerata. Arthropod structure & development, 39(2), 124-142.

Dunlop, J. A., Anderson, L. I., & Braddy, S. J. (2003). A redescription of *Chasmataspis laurencii* Caster & Brooks, 1956 (Chelicerata: Chasmataspidida) from the Middle Ordovician of Tennessee, USA, with remarks on chasmataspid phylogeny. Transactions of the Royal Society of Edinburgh: Earth Sciences, 94(03), 207-225.

Dunlop, J. A., & Arango, C. P. (2005). Pycnogonid affinities: A review. Journal of Zoological Systematics and Evolutionary Research, 43(1), 8-21.

Dunlop, J. A., Tetlie, E. O., & Prendini, L. (2008). Reinterpretation of the Silurian scorpion *Proscorpius osborni* (Whitfield): integrating data from Palaeozoic and Recent scorpions. Palaeontology, 51(2), 303-320.

Dunlop, J. A., & Webster, M. (1999). Fossil evidence, terrestrialization and arachnid phylogeny. Journal of Arachnology, 86-93.

Evans, G. O. (1992). *Principles of acarology*. CAB international.

Fahrenbach, W. H., & Arango, C. P. (2007). Microscopic anatomy of Pycnogonida: II. Digestive system. III. Excretory system. Journal of morphology, 268(11), 917-935.

Farley, R. D. (2005). Developmental changes in the embryo, pronymph, and first molt of the scorpion Centruroides vittatus (Scorpiones: Buthidae). Journal of morphology, 265(1), 1-27.

Firstman, B. (1973). The relationship of the chelicerate arterial system to the evolution of the endosternite. Journal of Arachnology, 1-54.

Foelix, R. F. (1985). Mechano-and chemoreceptive sensilla. In Neurobiology of arachnids (pp. 118-137). Springer Berlin Heidelberg.

Garwood, R. J., Sharma, P. P., Dunlop, J. A., & Giribet, G. (2014). A Paleozoic Stem Group to Mite Harvestmen Revealed through Integration of Phylogenetics and Development. Current Biology, 24(9), 1017-1023.

Giribet, G., Edgecombe, G. D., Wheeler, W. C., & Babbitt, C. (2002). Phylogeny and systematic position of opiliones: A combined analysis of chelicerate relationships using morphological and molecular data1. Cladistics, 18(1), 5-70.

Hammen, L. V. D. (1982). Comparative studies in chelicerata II. Epimerata (Palpigradi and Actinotrichida). Zoologische Verhandelingen, 196, 1-70.

Hammen, L. V. D. (1989). An introduction to comparative arachnology. SPB Academic Publishing bv.

Hara, M. R., & Gnaspini, P. (2003). Comparative study of the defensive behavior and morphology of the gland opening area among harvestmen (Arachnida, Opiliones, Gonyleptidae) under a phylogenetic perspective. Arthropod structure & development, 32(2), 257-275.

Harvey, M. S. (1992). The phylogeny and classification of the Pseudoscorpionida (Chelicerata: Arachnida). Invertebrate Systematics, 6(6), 1373-1435.

Homann, H. (1985). Die Cheliceren der Araneae, Amblypygi und Uropygi mit den Skleriten, den Plagulae (Chelicerata, Arachnomorpha). Zoomorphology, 105(2), 69-75.

Hou, X., & Bergström, J. (1997). Arthropods of the lower Cambrian Chengjiang fauna, southwest China. Fossils Strata, 45: 1-116.

Jeram, A. J. (2001). Paleontology. Scorpion biology and research, 370-392. Edyted by Brownell P, Polis G A, Oxford, Oxford

University Press, 2001

Jesionowska, K. (2003). Observations on the morphology of some eupodoid and endeostigmatic gnathosomata (Acariformes, Actinedida, Eupodoidea and Endeostigmata). Acta zoologica cracoviensia, 46(3), 257-268.

Kamenz, C., Dunlop, J. A., Scholtz, G., Kerp, H., & Hass, H. (2008). Microanatomy of Early Devonian book lungs. Biology letters, 4(2), 212-215.

Kethley, J. (1990). Acarina: Prostigmata (Actinedida). Soil biology guide, 1, 667-756. Dindal DL (ed), A Wiley -Interscience Publication, John Wiley & Sons, New York, USA.

King, P. E. ( 1973). *Pycnogonids*. Hutchinson University Library.

Klompen, J. S. H. (2000). Prelarva and larva of Opilioacarus (Neocarus) texanus (Chamberlin and Mulaik)(Acari: Opilioacarida) with notes on the patterns of setae and lyrifissures. Journal of Natural History, 34(10), 1977-1992.

Kraus, O. (1976). Zur phylogenetischen Stellung und Evolution der Chelicerata. Entomologica Germanica, 3, 1-12.

Kühl, G., Bergmann, A., Dunlop, J., Garwood, R. J., & Rust, J. (2012). Redescription and palaeobiology of *Palaeoscorpius devonicus* Lehmann, 1944 from the Lower Devonian Hunsrück Slate of Germany. Palaeontology, 55(4), 775-787.

Legg, D. A., Garwood, R. J., Dunlop, J. A., & Sutton, M. D. (2012). A taxonomic revision of orthosternous scorpions from the English Coal Measures aided by x-ray micro-tomography (XMT). Palaeontologia Electronica, 15(1), 1-16.

Legg, D. A., Sutton, M. D., & Edgecombe, G. D. (2013). Arthropod fossil data increase congruence of morphological and molecular phylogenies. Nature communications, 4, 2485.

Lindquist, E. E. (1984). Current theories on the evolution of major groups of Acari and on their relationships with other groups of Arachnida, with consequent implications for their classification. Acarology VI/editors, DA Griffiths and CE Bowman.

Michalik, P., & Alberti, G. (2005). On the occurrence of the 9+ 0 axonemal pattern in the spermatozoa of sheetweb spiders (Araneae, Linyphiidae). Journal of Arachnology, 33(2), 569-572.

Moore, R. A., Briggs, D. E., & Bartels, C. (2005). A new specimen of *Weinbergina opitzi* (Chelicerata: Xiphosura) from the Lower Devonian Hunsrück Slate, Germany. Paläontologische Zeitschrift, 79(3), 399-408.

Moritz, M. (1959). Zur Embryonalentwicklung der Phalangiiden (Opiliones; Palpatores) II. Die Anlage und Entwicklung der Coxaldrüse bei *Phalangium opilio* L. Zoologische Jahrbucher. Abtheilung für Anatomie und Ontogenie der Thiere, 77, 229-240.

Murienne, J., Harvey, M. S., & Giribet, G. (2008). First molecular phylogeny of the major clades of Pseudoscorpiones (Arthropoda: Chelicerata). Molecular phylogenetics and evolution, 49(1), 170-184.

Orr, P. J., Siveter, D. J., Briggs, D. E., Siveter, D. J., & Sutton, M. D. (2000). A new arthropod from the Silurian Konservat–Lagerstätte of Herefordshire, UK. Proceedings of the Royal Society of London. Series B: Biological Sciences, 267(1452), 1497-1504.

Pepato, A. R., da Rocha, C. E., & Dunlop, J. A. (2010). Phylogenetic position of the acariform mites: sensitivity to homology assessment under total evidence. BMC Evolutionary Biology, 10(1), 235.

Pinto-da-Rocha, R., Machado, G., & Giribet, G. (Eds.). (2007). Harvestmen: the biology of Opiliones. Harvard University Press.

Punzo, F. (1998). Biology of camel-spiders (Arachnida, Solifugae). Kluwer Academic Publishers Boston USA.

Reddell, J. R., & Cokendolpher, J. C. (1995). Catalogue, bibliography, and generic revision of the order Schizomida (Arachnida). Texas Memorial Museum Monologues 4, 1-170.

Roewer, C. F. (1934). Solifugae, palpigradi. In Klassen und Ordnungen des Tierreichs 5: Arthropoda. IV: Arachnoidea. Edited by Bronns H G, Leipzig, Akademische Verlagsgesellschaft M.B.H., 1934, 5(IV)(4)(4–5): 481–723

Rowland, J. M., & Cooke, J. A. (1973). Systematics of the arachnid order Uropygida (= Thelyphonida). Journal of Arachnology, 55-71.

Scholl, G. (1977). Beiträge zur Embryonalentwicklung von *Limulus polyphemus* L. (Chelicerata, Xiphosura). Zoomorphologie, 86(2), 99-154.

Scholtz, G., & Kamenz, C. (2006). The book lungs of Scorpiones and Tetrapulmonata (Chelicerata, Arachnida): evidence for homology and a single terrestrialisation event of a common arachnid ancestor. Zoology, 109(1), 2-13.

Selden, P. A. (1981). Functional morphology of the prosoma of Baltoeurypterus tetragonophthalmus (Fischer)(Chelicerata: Eurypterida). Transactions of the Royal Society of Edinburgh: Earth Sciences, 72(01), 9-48.

Selden, P. A., Shear, W. A., & Bonamo, P. M. (1991). A spider and other arachnids from the Devonian of New York, and reinterpretations of Devonian Araneae. Palaeontology, 34, 241-281.

Selden, P. A., Shear, W. A., & Sutton, M. D. (2008). Fossil evidence for the origin of spider spinnerets, and a proposed arachnid order. Proceedings of the National Academy of Sciences, 105(52), 20781-20785.

Shatrov, A. B. (2005). Ultrastructural investigations of the salivary glands in adults of the microtrombidiid mite *Platytrombidium fasciatum* (CL Koch, 1836)(Acariformes: Microtrombidiidae). Arthropod Structure & Development, 34(1), 49-61.

Shear, W. A., Selden, P. A., Rolfe, W. I., Bonamo, P. M., & Grierson, J. D. (1987). New terrestrial arachnids from the Devonian of Gilboa, New York (Arachnida, Trigonotarbida). American Museum Novitates, 2901, 1-74

Shultz, J. W. (1989). Morphology of locomotor appendages in Arachnida: evolutionary trends and phylogenetic implications. Zoological Journal of the Linnean Society, 97(1), 1-55.

Shultz, J. W. (1990). Evolutionary morphology and phylogeny of Arachnida. Cladistics, 6(1), 1-38.

Shultz, J. W. (1993). Muscular anatomy of the giant whipscorpion *Mastigoproctus giganteus* (Lucas)(Arachnida: Uropygi) and its evolutionary significance. Zoological Journal of the Linnean Society, 108(4), 335-365.

Shultz, J. W. (1999). Muscular anatomy of a whipspider, Phrynus longipes (Pocock)(Arachnida: Amblypygi), and its evolutionary significance. Zoological Journal of the Linnean Society, 126(1), 81-116.

Shultz, J. W. (2000). Skeletomuscular anatomy of the harvestman Leiobunum aldrichi (Weed, 1893) (Arachnida: Opiliones: Palpatores) and its evolutionary significance. Zoological Journal of the Linnean Society, 128(4), 401-438.

Shultz, J. W. (2001). Gross muscular anatomy of Limulus polyphemus (Xiphosura, Chelicerata) and its bearing on evolution in the Arachnida. Journal of Arachnology, 283-303.

Shultz, J. W. (2007a). A phylogenetic analysis of the arachnid orders based on morphological characters. Zoological Journal of the Linnean Society, 150(2), 221-265.

Shultz, J. W. (2007b). Morphology of the prosomal endoskeleton of Scorpiones (Arachnida) and a new hypothesis for the evolution of cuticular cephalic endoskeletons in arthropods. Arthropod structure & development, 36(1), 77-102.

Shultz, J. W., & Pinto-da-Rocha, R. (2007). Morphology and functional anatomy. Harvestmen: the Biology of Opiliones. Harvard University Press, Cambridge, 14-61.

Simonnet, F., Célérier, M. L., & Quéinnec, E. (2006). Orthodenticle and empty spiracles genes are expressed in a segmental pattern in chelicerates. Development genes and evolution, 216(7-8), 467-480.

Snodgrass, R. E. (1948). The feeding organs of Arachnida, including mites and ticks (Vol. 3944). Smithsonian Institution Misc Collection, 110, 1-93.

Stein, M., & Selden, P. A. (2012). A restudy of the Burgess Shale (Cambrian) arthropod *Emeraldella brocki* and reassessment of its affinities. Journal of Systematic Palaeontology, 10(2), 361-383.

Storch, V., Alberti, G., & Ruhberg, H. (1979). Light and electron microscopical investigations on the salivary glands of *Opisthopatus cinctipes* and *Peripatopsis mosleyi* (Onychophora: Peripatopsidae). Zoologischer Anzeiger, 203(1-2), 35-47.

Stürmer, W., & Bergström, J. (1981). *Weinbergina*, a xiphosuran arthropod from the Devonian Hunsrück Slate. Paläontologische Zeitschrift, 55(3-4), 237-255.

Sutton, M. D., Briggs, D. E., Siveter, D. J., Siveter, D. J., & Orr, P. J. (2002). The arthropod *Offacolus kingi* (Chelicerata) from the Silurian of Herefordshire, England: computer based morphological reconstructions and phylogenetic affinities. Proceedings of the Royal Society of London. Series B: Biological Sciences, 269(1497), 1195-1203.

Talarico, G., Palacios-Vargas, J. G., Silva, M. F., & Alberti, G. (2005). First ultrastructural observations on the tarsal pore organ of Pseudocellus pearsei and P. boneti (Arachnida, Ricinulei). Journal of Arachnology, 33(2), 604-612.

Thomas, R. H., & Telford, M. J. (1999). Appendage development in embryos of the oribatid mite Archegozetes longisetosus (Acari, Oribatei, Trhypochthoniidae). Acta Zoologica, 80(3), 193-200.

Vilpoux, K., & Waloszek, D. (2003). Larval development and morphogenesis of the sea spider *Pycnogonum litorale* (Ström, 1762) and the tagmosis of the body of Pantopoda. Arthropod structure & development, 32(4), 349-383.

Walossek, D., & Müller, K. J. (1998). Cambrian ‘Orsten’-type arthropods and the phylogeny of Crustacea. In Arthropod relationships (pp. 139-153). Springer Netherlands.

Walter, D. E., & Proctor, H. C. (1998). Feeding behaviour and phylogeny: observations on early derivative Acari. Experimental & applied acarology, 22(1), 39-50.

Weygoldt, P. (1969). Biology of pseudoscorpions. Harvard University Press Cambridge USA.

Weygoldt, P. (1978). Paarungsverhalten und Spermatophorenmorphologie bei Geißelskorpionen: *Thelyphonellus amazonicus* Butler und *Typopeltis crucifer* Pocock (Arachnida, Uropygi). Zoomorphologie, 89(2), 145-156.

Weygoldt, P. (1999). Evolution and systematics of the Chelicerata. In *Ecology and Evolution of the Acari* (pp. 1-14). Springer Netherlands.

Weygoldt, P., & Paulus, H. F. (1979). Untersuchungen zur Morphologie, Taxonomie und Phylogenie der Chelicerata1 II. Cladogramme und die Entfaltung der Chelicerata. Journal of Zoological Systematics and Evolutionary Research, 17(3), 177-200.

Weygoldt, P. (2000). Whip spiders (Chelicerata: Amblypygi): their biology, morphology and systematics. ISBS.

Wheeler, W. C., & Hayashi, C. Y. (1998). The phylogeny of the extant chelicerate orders. Cladistics, 14(2), 173-192.

Wills, M. A., Briggs, D. E., Fortey, R. A., Wilkinson, M., & Sneath, P. H. (1998). An arthropod phylogeny based on fossil and recent taxa. Arthropod fossils and phylogeny. Columbia University Press, New York, 33-105.

Winkler, D. (1957). Die Entwicklung der äusseren Körpergestalt bei den Phalangiidae (Opiliones). Mitteilungen aus dem Museum für Naturkunde in Berlin. Zoologisches Museum und Institut für Spezielle Zoologie (Berlin), 33(2), 355-389.

Yoshikura, M. (1975). Comparative embryology and phylogeny of Arachnida. Kumamoto journal of science, Biology, 12(2), 71-142.

Zacharda, M. (1980). Soil mites of the family Rhagidiidae (Actinedida: Eupodoidea): Morphology, systematics, ecology. Acta Universitatis Carolinae Biologica, 5-6, 489-785.

Zakhvatkin, A. A. (1949). New representatives of apparently segmented mites (Acarina: Pachygnathidae). Ent. Obozer. Moscow, 30, 291-295.
